# A multiscale sliding filament model of lymphatic muscle pumping

**DOI:** 10.1007/s10237-021-01501-0

**Published:** 2021-09-02

**Authors:** Christopher J. Morris, David C. Zawieja, James E. Moore

**Affiliations:** 1 Department of Bioengineering, Imperial College London, South Kensington Campus, London, SW7 2AZ UK; 2 College of Medicine Faculty, Texas A&M University, Texas, USA

**Keywords:** Lymphatics, Muscle, Multiscale model, Sliding filament model, Lymphedema

## Abstract

The lymphatics maintain fluid balance by returning interstitial fluid to veins via contraction/compression of vessel segments with check valves. Disruption of lymphatic pumping can result in a condition called lymphedema with interstitial fluid accumulation. Lymphedema treatments are often ineffective, which is partially attributable to insufficient understanding of specialized lymphatic muscle lining the vessels. This muscle exhibits cardiac-like phasic contractions and smooth muscle-like tonic contractions to generate and regulate flow. To understand the relationship between this sub-cellular contractile machinery and organ-level pumping, we have developed a multiscale computational model of phasic and tonic contractions in lymphatic muscle and coupled it to a lymphangion pumping model. Our model uses the sliding filament model (Huxley in Prog Biophys Biophys Chem 7:255–318, 1957) and its adaptation for smooth muscle (Mijailovich in Biophys J 79(5):2667–2681, 2000). Multiple structural arrangements of contractile components and viscoelastic elements were trialed but only one provided physiologic results. We then coupled this model with our previous lumped parameter model of the lymphangion to relate results to experiments. We show that the model produces similar pressure, diameter, and flow tracings to experiments on rat mesenteric lymphatics. This model provides the first estimates of lymphatic muscle contraction energetics and the ability to assess the potential effects of sub-cellular level phenomena such as calcium oscillations on lymphangion outflow. The maximum efficiency value predicted (40%) is at the upper end of estimates for other muscle types. Spontaneous calcium oscillations during diastole were found to increase outflow up to approximately 50% in the range of frequencies and amplitudes tested.

**Supplementary Information:**

The online version contains supplementary material available at 10.1007/s10237-021-01501-0.

## Introduction

Fluid homeostasis is maintained by the converging vessels of the lymphatic system that in humans return 4–8 L of interstitial fluid to the venous system in lymph nodes and at the subclavian veins. Lymphatics lack a central pump equivalent to the heart, so fluid propulsion is achieved by a combination of active lymphatic contraction and external compression from surrounding tissues. The relative contributions of intrinsic contractions and external compression vary throughout the lymphatic tree. The initial lymphatic vessels, consisting of endothelial lining and basement membrane, do not actively contract in most tissue and species. These vessels lead to larger “collecting” lymphatic vessels, where active contractions often occur due to specialized lymphatic muscle cells (LMCs) in the wall. Retrograde flow in collecting lymphatics is minimized by closely spaced one-way intraluminal valves (Gashev 2008; Margaris & Black 2012; Moore Jr. & Bertram 2018). Vessel contractions are very nearly uniform in the segments between valves (called “lymphangions”), so peristalsis is not a relevant mechanism for active pumping. In many anatomical locations, lymph transport works against an adverse pressure gradient to remove fluid from low or subatmospheric pressures in the interstitium into and along lymphatics with higher positive pressures (Zweifach and Prather 1975, Hargens and Zawiefach 1977, Aukland & Reed 1993; Guyton et al. 1971; Jamalian et al. 2017).

Deficiencies in lymph transport can result in a chronic, debilitating condition involving tissue swelling from accumulation of interstitial fluid and proteins called lymphedema. The effectiveness of lymphedema management strategies is limited, so it is generally said that it has no cure (Fu 2014). The absence of lymphedema treatments can be attributed in part to a lack of understanding of LMC contraction dynamics (Scallan et al. 2016; Zhang et al. 2013).

The dual roles of lymphatic vessels serving as both pumps and conduits mean that intrinsic contractions from LMCs must perform the tasks of both cardiac muscle (rapid, short-lasting phasic contractions to generate flow) and vascular smooth muscle (slower, long-lasting tonic constriction to regulate flow through diameter-based resistance) (Bridenbaugh et al. 2003; Quick et al. 2007; von der Weid & Zawieja 2004). Lymphatic muscle contractions can adapt in the manner of a heart or a resistance artery, depending on the local lymphodynamic environment (Gashev et al. 2004, 2012).

The dual behaviors of LMCs are grounded in the presence of two classes of contractile and regulatory proteins. These cells possess both striated (slow twitch β) and phasic smooth (SM1B and SM2B) muscle myosin heavy chain isoforms as well as four different actin proteins from both smooth and striated muscle types—cardiac α, skeletal α, vascular α and enteric γ (Muthuchamy et al. 2003). The key difference between heavy chain isoforms is that tonic myosin heads can enter a slowly cycling latch state (Murphy & Rembold 2005; Seow 2016) typically observed in smooth muscles. Similar to other types of muscle cells, the excitation of LMCs results from spontaneous depolarizations arising at pacemaker sites within the muscle layer (von der Weid 2001; von der Weid & Zawieja 2004; Zawieja et al. 1999). These depolarizations can sum to generate action potentials which then cause rapid increases in the intracellular calcium concentration (von der Weid et al. 2014; Zawieja et al. 1999). In smooth muscle cells, various factors including membrane depolarization, stretch/strain, and membrane receptor activation will also lead to elevations in intracellular calcium concentration. Thus, in both smooth and striated muscle types, changes in intracellular calcium lead to activation of the muscle regulatory proteins that produce the actin-myosin interactions that contract the muscle cell.

For striated muscle, calcium binding to troponin C (TnC) the calcium-sensitive portion of the troponin complex causes a conformation change in the troponin-tropomyosin physical interaction. This shifts the interactions of tropomyosin and actin and exposes actin sites for myosin head attachment. Troponin T and I have been shown in lymphatic muscle (Zolla et al. 2015), and there is preliminary evidence for the presence of slow/cardiac TnC in lymphatic muscle (unpublished observation of D. Zawieja). It is generally accepted that the primary means of excitation–contraction coupling (ECC) for smooth muscle is calcium binding to calmodulin (CaM). This activates myosin light chain kinase to phosphorylate myosin heads, allowing binding of myosin to actin sites and force generation. Tonic contraction of LMCs is dependent on the balance of phosphorylation of the myosin light chain produced by the opposing effects of myosin light chain kinase activation by binding of calcium to CaM and myosin light chain phosphatase (Dougherty et al. 2008; Wang et al. 2009).

No model for the subcellular mechanisms of lymphatic muscle contractility exists. Existing models of lymphangions prescribe the intrinsic contraction forces. Computational research into lymphatic muscle has focused on the regulation of contractions and the electrical properties of lymphatic muscle (Baish et al. 2016; Contarino & Toro 2018; Kunert et al. 2015). While there have been multiple computer models phenomenologically incorporating both phasic and tonic contractions of lymphatic muscle (Caulk et al. 2016; Caulk et al. 2015; Kunert et al. 2015; Razavi et al. 2020; Razavi et al. 2017), the different effects of the contraction types were the focus of only one series of computational modelling papers. This model used the time-varying elastance model of the heart combined with the transmission line equations for blood vessels (Quick et al. 2008; Venugopal et al. 2007; Venugopal et al. 2010; Venugopal et al. 2004; Venugopal et al. 2003).

While this model was important in developing an understanding of the hybrid nature of lymphatic muscle, it did not incorporate the subcellular mechanisms within the muscle that give rise to the dual functional behavior. The phenomenological nature of these models combined with the fact that they do not explicitly model the subcellular components limits their applicability in studying the effects of modulating subcellular components in health and disease.

Many muscle models are based on those of A.F. Huxley (Huxley 1957) or of A.V. Hill (Hill 1938). Huxley’s model is a mechanistic model of the molecular interactions of actin and myosin filaments of muscle (described in more detail below). Hill’s model is a black-box model that relates velocity and force generation. Hill’s model lacks a direct relationship between the mechanical and metabolic behaviors (Huxley’s includes such a relationship). Computational intensity initially hindered use of the Huxley model but this has become less of an issue (Lemaire et al. 2016).

In this paper, we develop a sub-cellular model of LMCs to gain an understanding of the interaction between contraction types and their influence on lymph pumping. The model’s performance is tested by integrating it within our previously validated lumped parameter model of lymphatic pumping (Bertram et al. 2017, 2014a, 2011, 2014b, 2016; Jamalian et al. 2013, 2016, 2017). We used existing experimental results obtained using rat mesenteric vessels as a basis for model parameter values.

## Methods

**Fig. 1:**
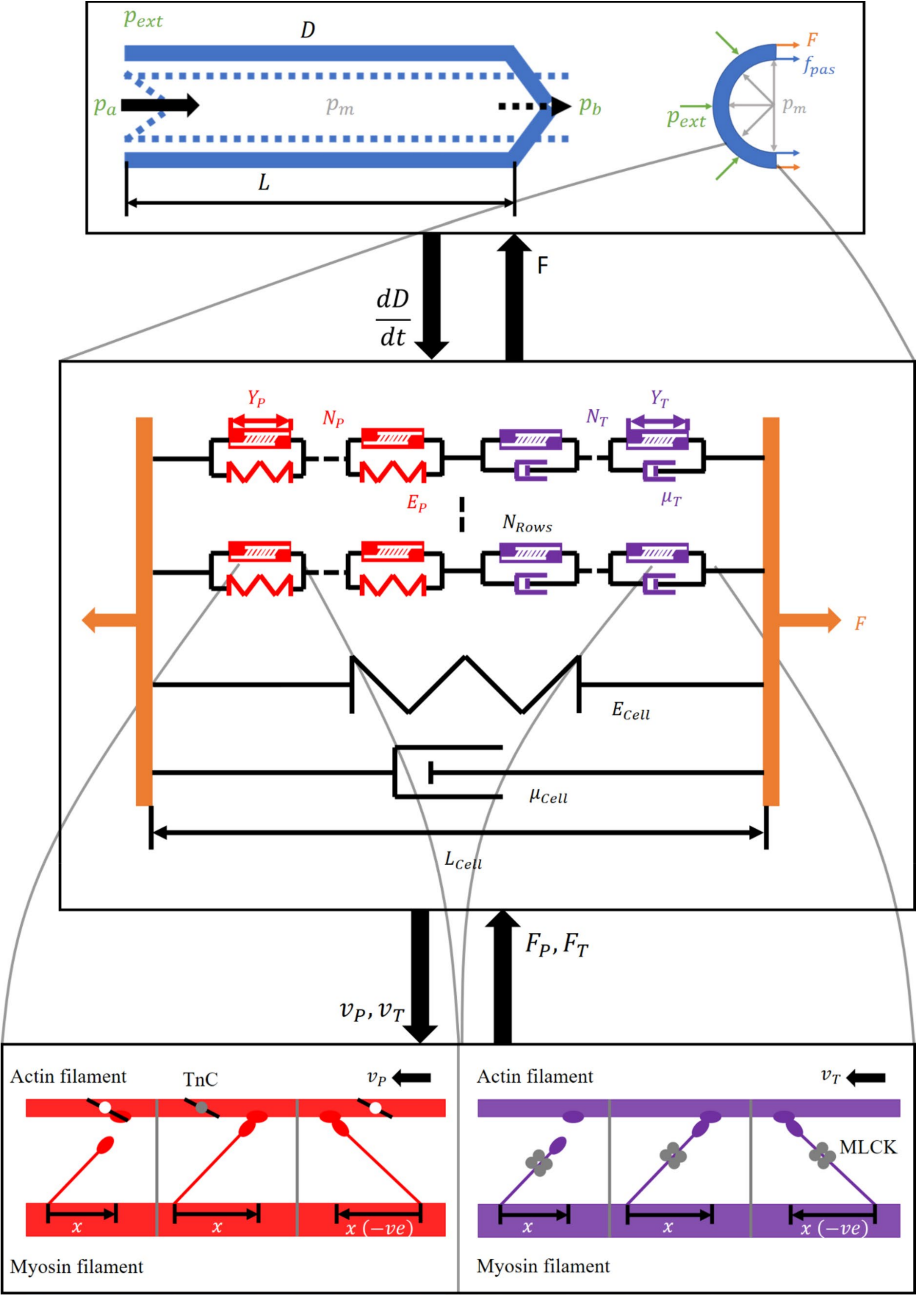
Coupling of scales. The largest scale in the model is based on a lumped parameter model of lymphangion pumping, which yields mesoscale pressure-flow relations. Intrinsic muscle contractions are incorporated through a wall-force balance to calculate the pressure in the center of a lymphangion. LMC contractile force is calculated from a model that includes two types of contractile elements (CEs) connected in series with a spring in parallel with phasic elements and dashpot in parallel with tonic elements. There is also a parallel viscoelasticity representing cell properties. The molecular force generation of CEs is calculated from the sliding filament model with ECC incorporated using a prescribed periodic function of intracellular free calcium concentration

The sub-cellular muscle model is based on the molecular sliding filament model of Huxley (Huxley 1957) and its adaptation for smooth muscle (Fredberg et al. 1999; Mijailovich et al. 2000). The fully coupled model consists of three scales: molecular, cellular, and lymphangion. Scales are coupled by iteratively passing down the velocity from a larger scale to a smaller scale and returning the contractile force, as shown by Fig. 1. The effects of ECC are included in the muscle model to induce periodic contractions. We base the model parameters on rat mesenteric collecting lymphatics unless otherwise specified. A full list of the symbols used in this paper is included in the Supplementary material.

### Cell model

The combination of tonic and phasic contractions is captured at the cell scale through two types of molecular contractile elements (CEs). Viscoelasticity of LMCs is modelled using a Kelvin–Voigt viscoelastic element in parallel with the CEs (Fig. 1), based on the work of Brook and Jensen (Brook & Jensen 2014). CEs provide a velocity- and calcium-dependent force modelled as described in Sect. 2.2.

CEs of both types are connected in series. A strain-stiffening spring is included in parallel with phasic CEs (referred to as phasic spring) and a Newtonian dashpot in parallel with tonic CEs (referred to as tonic dashpot). The phasic spring allows the tonic force during diastole to be transmitted across passive or minimally active phasic CEs and affect the cell force. This is necessary to allow tonic constriction of the lymphangion. The tonic dashpot allows phasic contractions to rapidly reduce the cell length without causing a significant change in tonic CE length. This is necessary to allow the tonic CEs to attach enough heads for physiologic force generation. In the development of this model, several series and parallel arrangements of CEs and mechanical elements were trialed, but this was the only arrangement that yielded physiologic behaviors. The other arrangements that were tried were parallel rows of CEs, series contractile units with passive elements (spring and dashpot) in parallel with phasic CEs only, and series CEs with passive elements in parallel with tonic CEs only. The key problem with the parallel arrangement was that the faster velocity of phasic CEs imposed that velocity on the tonic CEs so the tonic CEs could not attach enough heads for physiologic force generation. Having passive elements in parallel with the phasic CEs meant that the over all force of the CE row was the tonic force (the passive elements simply made up the difference so that the combination of phasic CEs and passive elements was the tonic force). Similarly, with passive elements in parallel with the tonic CEs, the row force was the phasic force.

A force balance between CEs with contributions from the phasic spring and tonic dashpot results in the following equation for the force developed by a single row $\left({F}_{\mathrm{Row}}\right)$ containing the CEs (Fig. 1).

1 $$ F_{{{\text{Row}}}} = F_{P} + E_{P} \left( {N_{P} Y_{P} - N_{P} Y_{{P,{\text{ref}}}} } \right) = F_{T} + \mu_{T} N_{T} \frac{{{\text{dY}}_{T} }}{{{\text{dt}}}} $$

where ${F}_{P}$ is the force generated by phasic CEs, ${E}_{P}$ is the stiffness of the phasic spring, ${N}_{P}$ is the number of phasic CEs in series, ${Y}_{P}$ is the length of phasic CEs, ${Y}_{P,\mathrm{ref}}$ is the reference length for zero force from the spring in parallel with phasic CEs, ${F}_{T}$ is the force generated by tonic CEs, ${\mu }_{T}$ is the viscosity constant for the tonic dashpot,${N}_{T}$ is the number of tonic CEs in series, ${Y}_{T}$ is the length of tonic CEs, and *t* is time.

The strain-stiffening of the phasic spring is given by the exponential equation

2 $$ E_{P} = ae^{{bN_{P} Y_{P} }} $$

where *a*, *b* are constitutive parameters.

It is essential for the phasic spring to be in extension during diastole when there is little phasic force, and tonic CEs pull on the phasic CEs. This means that the phasic spring needs to be resisting extension to prevent overextension of phasic CEs. Both types of CE are initialized to the same length (see Sect. 2.3).

The total combined length of phasic and tonic CEs corresponds to the cell length $\left({L}_{\mathrm{Cell}}\right)$

3 $$ L_{{{\text{Cell}}}} = N_{P} Y_{P} + N_{T} Y_{T} $$

The total contractile force of the cell $\left(F\right)$ is the sum of the over all parallel contributions

4 $$ F = N_{{{\text{Rows}}}} F_{{{\text{Row}}}} + E_{{{\text{Cell}}}} \left( {L_{{{\text{Cell}}}} - L_{{\text{Cell,ref}}} } \right) + \mu_{{{\text{Cell}}}} \frac{{{\text{dL}}_{{{\text{Cell}}}} }}{{{\text{dt}}}} $$

where ${N}_{\mathrm{Rows}}$ is the number of parallel rows of CEs, ${E}_{\mathrm{Cell}}$ is the stiffness of the LMC, ${L}_{\mathrm{Cell},\mathrm{ref}}$ is the length of an LMC with zero force from the cell stiffness, and ${\mu }_{\mathrm{Cell}}$ is the viscosity constant for the LMC.

The shortening velocity of tonic CEs $\left({v}_{T}\right)$ is obtained by rearranging the series force balance (Eq. 1), and the shortening velocity of phasic CEs $\left({v}_{P}\right)$ by differentiating and rearranging the length balance (Eq. 3)

5a $$ v_{T} = - \frac{{{\text{dY}}_{T} }}{{{\text{dt}}}} = \frac{{F_{P} + E_{P} \left( {N_{P} Y_{P} - N_{P} Y_{{P,{\text{ref}}}} } \right) - F_{T} }}{{\mu_{T} N_{T} }} $$

5b $$ v_{P} = - \frac{{{\text{dY}}_{P} }}{{{\text{dt}}}} = \frac{{{\text{dL}}_{{{\text{Cell}}}} /{\text{dt}} - N_{T} {\text{dY}}_{T} /{\text{dt}}}}{{N_{P} }} $$

### Molecular muscle modelling

The molecular models for CE force generation are based on adapting the sliding filament model to include the effects of ECC. In the sliding filament model, myosin heads are modelled as linearly elastic springs (Huxley 1957) and can occupy different chemical states. Each myosin head has its own equilibrium position that moves with the myosin filament, and its contractile force when attached is proportional to the displacement from equilibrium (Fig. 1). The maximum displacement at which a myosin head can still attach to actin is called the powerstroke length (assumed consistent between myosin isoforms). Detachment is possible at all displacements. We assume that there is an actin site:myosin head ratio of 1:1 and sufficient spacing between actin sites that each myosin head has only one actin site in attachment range. In phasic CEs, excitation–contraction coupling is included via troponin which affects the actomyosin attachment rate (Wong 1971, 1972). In tonic CEs, ECC is included via CaM which affects the myosin phosphorylation rate to drive actomyosin attachment (Wang et al. 2008; Yochum et al. 2015).

**Fig. 2:**
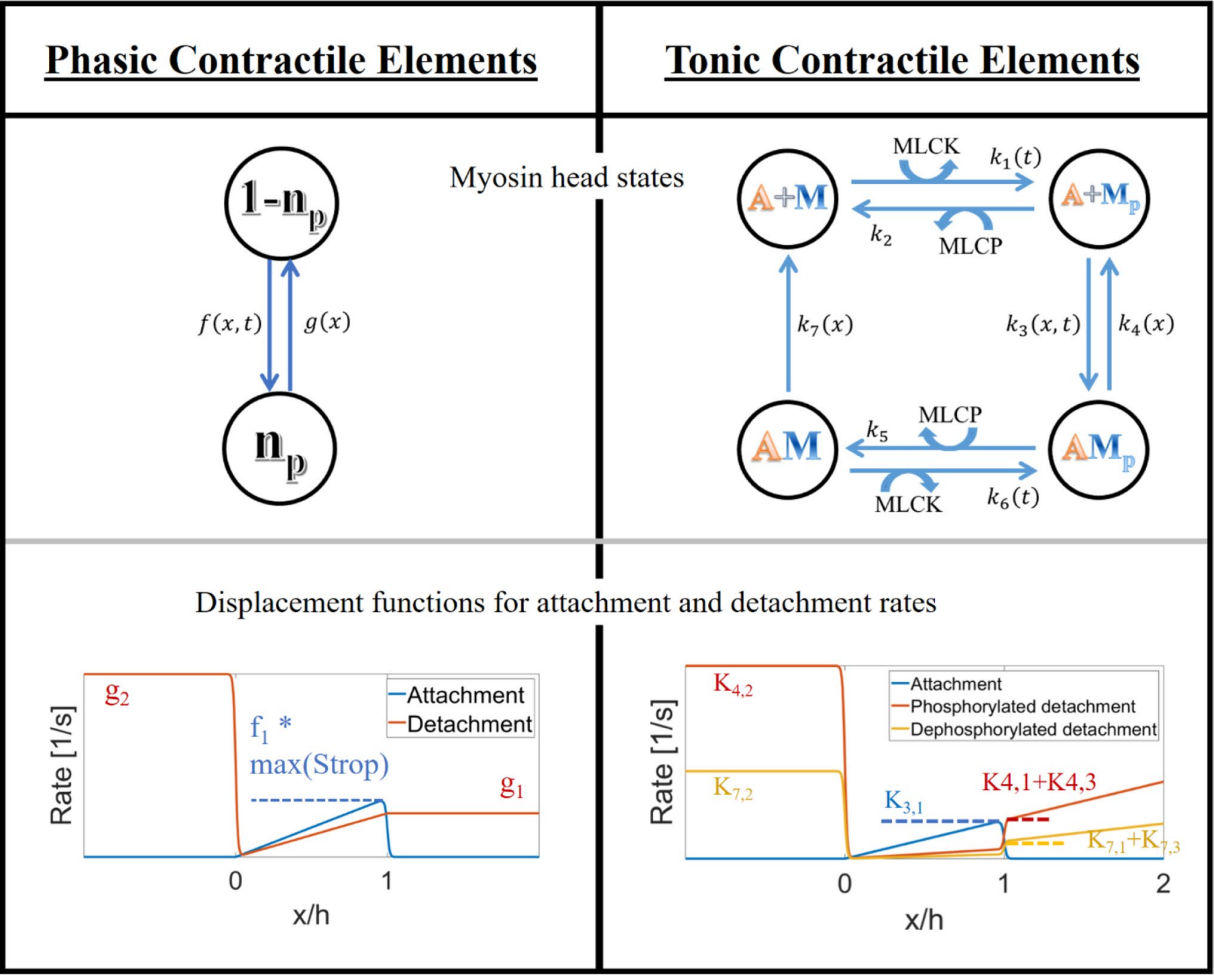
Molecular models of phasic and tonic CE myosin states (upper row) and myosin head rates (lower row)

It is assumed that phasic myosin heads can occupy two states with the head either attached and generating force or detached (see Fig. 2). The probability of a myosin head attaching $\left({\varvec{f}}\right)$ or detaching $\left({\varvec{g}}\right)$ is dependent on its displacement using the functions of A.F. Huxley (Huxley 1957) modified so that the detachment probability is constant for positive displacements greater than the powerstroke length. Constants for transition rates of both myosin isoforms are listed in Table 1. In the model presented here, the attachment rate is also dependent on the saturation of cardiac TnC with calcium ions, modelled as described in Sect. 2.2.1. Continuous gradients of the rate function were ensured by making the transitions part sines.

6a $$ f = \left\{ \begin{gathered} 0\quad \quad \quad \quad \quad \quad \quad \quad \quad \quad \quad \quad \quad \quad {\text{if}}\;x < 0 \hfill \\ S_{{{\text{Trop}}}} f_{1} x/h\quad \quad \quad \quad \quad \quad \quad \quad \quad \quad {\text{if}}\;0 \le x < h - {\text{dx}} \hfill \\ S_{{{\text{Trop}}}} f_{1} 0.9\left( {\sin \left( {\frac{2\pi x}{{0.4h}}} \right)/2 + 0.5} \right)\quad \quad {\text{if}}\;h - {\text{dx}} \le x \le h + {\text{dx}} \hfill \\ 0\quad \quad \quad \quad \quad \quad \quad \quad \quad \quad \quad \quad \quad \quad {\text{if}}\;x > h + {\text{dx}} \hfill \\ \end{gathered} \right. $$

where ${S}_{\mathrm{Trop}}$ is the saturation of TnC, ${f}_{1}$ is a constant, $x$ is displacement, and $h$ is powerstroke length

6b $$ g = \left\{ \begin{gathered} g_{2} \quad \quad \quad \quad \quad \quad \quad \quad \quad \quad \quad \quad \quad \quad \quad \quad \quad \quad \quad {\text{if}}\; x < - {\text{dx}} \hfill \\ \left( {g_{2} - 0.1g_{1} } \right)\left( {\sin \left( {\frac{2\pi x}{{0.4h}} - \pi } \right)/2 + 0.5} \right) + 0.1g_{1} \quad \quad {\text{if}}\; - {\text{dx}} \le x \le {\text{dx}} \hfill \\ g_{1} x/h\quad \quad \quad \quad \quad \quad \quad \quad \quad \quad \quad \quad \quad \quad \quad \quad \quad {\text{if}}\; {\text{dx}} < x \le h \hfill \\ g_{1} \quad \quad \quad \quad \quad \quad \quad \quad \quad \quad \quad \quad \quad \quad \quad \quad \quad \quad \quad {\text{if}}\; x > h \hfill \\ \end{gathered} \right. $$

where ${g}_{1},{g}_{2}$ are constants.

**Table 1:** Rate constants used for phasic and tonic myosin head state transitions

Constant symbol	Value [1/s]	Source
$f_{1}$ $g_{1}$ $g_{2}$	620 50 210	Fit to general features (pressure, diameter, flow) of rat mesenteric lymphangion experiments (Davis et al. 2008; Davis et al. 2011; Davis et al. 2012; Scallan et al. 2016)
$K_{3,1}$ $K_{4,1}$ $K_{4,2}$ $K_{4,3}$ $K_{7,1}$ $K_{7,2}$ $K_{7,3}$ $K_{1}$ $K_{2}$ $K_{5}$ $K_{6}$	0.88 0.22 4($K_{3,1} + K_{4,1}$) 3 $K_{4,1}$ 0.1 20 $K_{7,1}$ 3 $K_{7,1}$ 0.35 0.1 K_2_ K_1_	Airway smooth muscle cells (Fredberg et al. 1999)

The displacement-distribution of attached phasic heads can be affected by either detachment or mechanical convection to a different displacement. Using first-order kinetics for myosin head state transitions, the attached proportion of myosin heads is governed by

7 $$ \frac{{\partial n_{P} }}{\partial t} - v_{P} \frac{{\partial n_{P} }}{\partial x} = \left( {1 - n_{P} } \right)f - n_{P} g $$

where ${n}_{P}$ is the proportion of phasic myosin heads with displacement $x$ that are attached, $f$ is the attachment rate, and $g$ is the detachment rate. Conservation of the total number of myosin heads dictates that the fraction of detached heads is $\left(1-{n}_{P}\right)$.

The force per phasic CE $\left({F}_{P}\right)$ is then calculated as in (Huxley 1957) by integrating Hooke’s law over all displacements with constants for the number of myosin heads and density of actin binding sites.

8 $$ F_{P} = \rho {\text{Num}}_{P} K_{P} \mathop \smallint \limits_{ - \infty }^{\infty } xn_{P} {\text{dx}} $$

where $\rho$ is the length density of actin binding sites along the thin filament, ${\mathrm{Num}}_{P}$ is the number of myosin heads in a phasic CE, and ${K}_{P}$ is phasic myosin head stiffness.

The integral was, in practice, bounded by finite values of − 15*h* to 15*h* which were sufficiently far from the attachment region that all heads were detached, so that no force contribution could result from increasing these bounds. Displacement discretization was twenty cells per powerstroke length with the sine region subdivided by ten (one displacement discretization on either side of the rate transitions at $x=0$ and $x=h$). See the Supplementary material for some plots showing negligible effects of halving the displacement discretization.

Tonic CE mechanics are also modelled using the sliding filament model, but with additional states motivated by the presence of the slowly cycling latch state of smooth muscle (Dillon et al. 1981; Murphy & Rembold 2005) (Fig. 2). The models for these additional states come from Hai and Murphy (Hai & Murphy 1988a, 1988b,^ 1992^). Tonic CE transition rate functions for attachment and detachment (Fig. 2) are similar to those for phasic CEs, except with increased detachment rates for heads with greater displacement than the powerstroke length (Brook & Jensen 2014). Transitions between the piecewise functions are again given by part sines.

The resulting set of partial differential equations describing the displacement-distribution of tonic heads in different states can be written as a single equation with a vector of attachment states and a matrix of transition rates (Fredberg et al. 1999). The force generated by tonic CEs $\left({F}_{T}\right)$ is given by the integral of attached states over all displacements (Brook & Jensen 2014) again with constants of the number of myosin heads and density of actin binding sites.

9 $$ F_{T} = \rho {\text{Num}}_{T} K_{T} \left( {\mathop \smallint \limits_{ - \infty }^{\infty } x \times AM_{p} {\text{dx}} + \mathop \smallint \limits_{ - \infty }^{\infty } x \times AM {\text{dx}}} \right) $$

where ${\mathrm{Num}}_{T}$ is the number of myosin heads in a tonic CE, ${K}_{T}$ is the stiffness of tonic myosin heads, ${AM}_{p}$ is the proportion of tonic myosin heads in the attached phosphorylated state, and $AM$ is the proportion of tonic myosin heads in the attached dephosphorylated state.

To analyze the system’s efficiency, the rate of energy liberation is calculated. One molecule of adenosine triphosphate (ATP) is hydrolyzed per detachment or phosphorylation process (in tonic CEs). The energy liberated by detachment is calculated from the myosin detachment rate and number of attached heads. The number of attached heads is given by the proportion of heads attached integrated over all displacements and multiplied by the density of actin sites. The total energy liberation rate is the product of the number of CE rows and the energy liberated from a single row, then summed over all LMCs.

10 $$ \frac{{{\text{dEnergy}}_{P} }}{{{\text{dt}}}} = N_{{{\text{Cell}}}} N_{{{\text{Rows}}}} \rho u{\text{Num}}_{P} N_{P} \mathop \smallint \limits_{ - \infty }^{\infty } gn_{P} {\text{dx}} $$

where ${\mathrm{Energy}}_{P}$ is the energy liberated by detachment of phasic myosin heads, ${N}_{\mathrm{Cell}}$ is the circumferential number of LMCs, and $u$ is the energy liberated by hydrolysis of one ATP molecule.

The rate of energy liberation in phasic CEs is due solely to the energy liberated in detachment. The energy liberation in tonic CEs is the sum of detachment and phosphorylation events (Mijailovich et al. 2000). The liberation rates here are summed over all CEs to give the energy liberated by each LMC and then summed over all LMCs.

11a $$ \frac{{{\text{dEnergy}}_{{T,{\text{PhosDetach}}}} }}{{{\text{dt}}}} = N_{{{\text{Cell}}}} \rho uN_{{{\text{Rows}}}} {\text{Num}}_{T} N_{T} \mathop \smallint \limits_{ - \infty }^{\infty } k_{4} AM_{p} {\text{dx}} $$

11b $$ \frac{{{\text{dEnergy}}_{{T,{\text{UnphosDetach}}}} }}{{{\text{dt}}}} = N_{{{\text{Cell}}}} \rho uN_{{{\text{Rows}}}} {\text{Num}}_{T} N_{T} \mathop \smallint \limits_{ - \infty }^{\infty } k_{7} AM {\text{dx}} $$

11c $$ \frac{{{\text{dEnergy}}_{{T,{\text{DetachPhos}}}} }}{{{\text{dt}}}} = N_{{{\text{Cell}}}} \rho uN_{{{\text{Rows}}}} {\text{Num}}_{T} N_{T} k_{1} \mathop \smallint \limits_{ - \infty }^{\infty } M {\text{dx}} $$

11d $$ \frac{{{\text{dEnergy}}_{{T,{\text{AttachPhos}}}} }}{{{\text{dt}}}} = N_{{{\text{Cell}}}} \rho uN_{{{\text{Rows}}}} {\text{Num}}_{T} N_{T} k_{6} \mathop \smallint \limits_{ - \infty }^{\infty } AM {\text{dx}} $$

11e $$ \frac{{{\text{dEnergy}}_{T} }}{{{\text{dt}}}} = N_{{{\text{Cell}}}} \rho uN_{{{\text{Rows}}}} {\text{Num}}_{T} N_{T} \left( {\mathop \smallint \limits_{ - \infty }^{\infty } k_{4} AM_{p} {\text{dx}} + \mathop \smallint \limits_{ - \infty }^{\infty } k_{7} AM {\text{dx}} + k_{1} \mathop \smallint \limits_{ - \infty }^{\infty } M {\text{dx}} + k_{6} \mathop \smallint \limits_{ - \infty }^{\infty } AM {\text{dx}}} \right) $$

where ${\mathrm{Energy}}_{T,\mathrm{PhosDetach}}$ is the energy liberated by detachment of phosphorylated tonic myosin heads, ${k}_{4}$ is the detachment rate of phosphorylated tonic heads, ${\mathrm{Energy}}_{T,\mathrm{UnphosDetach}}$ is the energy liberated by detachment of unphosphorylated myosin heads, ${k}_{7}$ is the detachment rate for dephosphorylated myosin heads, ${\mathrm{Energy}}_{T,\mathrm{DetachPhos}}$ is the energy liberated by phosphorylation of detached myosin heads, ${k}_{1}$ is the phosphorylation rate for detached myosin heads, $M$ is the proportion of myosin heads in the detached dephosphorylated state, ${\mathrm{Energy}}_{T,\mathrm{AttachPhos}}$ is the energy liberated by phosphorylation of attached myosin heads, ${k}_{6}$ is the phosphorylation rate for attached myosin heads, and ${Energy}_{T}$ is the total energy liberated by tonic CEs.

The rate of doing work per LMC, $\mathrm{dW}/\mathrm{dt}$, is given by the product of force and shortening velocity, which is then summed over all cells.

12 $$ \frac{{{\text{dW}}}}{{{\text{dt}}}} = - \frac{{{\text{dL}}_{{{\text{Cell}}}} }}{{{\text{dt}}}}N_{{{\text{Cell}}}} F $$

The total energy liberated and work done comes from integrating their rates over the duration of a cycle. The over all energy liberated by LMCs is the sum of energy liberation by phasic and tonic CEs. Whatever liberated energy not used to do useful work is lost as heat. The energy efficiency is the useful work done divided by the energy liberated.

Energy loss rates due to the viscous elements of the subcellular model are calculated as

13a $$ \frac{{{\text{dE}}_{{{\text{TDloss}}}} }}{{{\text{dt}}}} = N_{{{\text{Rows}}}} N_{{{\text{Cell}}}} \mu_{T} \left( {N_{T} \frac{{{\text{dY}}_{HHM} }}{{{\text{dt}}}}} \right)^{2} $$

where ${E}_{\mathrm{TDloss}}$ is the loss of energy to viscsosity of the tonic dashpot

13b $$ \frac{{{\text{dE}}_{{{\text{CSloss}}}} }}{{{\text{dt}}}} = N_{{{\text{Cell}}}} \mu_{{{\text{Cell}}}} \left( {\frac{{{\text{dL}}_{{{\text{Cell}}}} }}{{{\text{dt}}}}} \right)^{2} $$

where ${E}_{\mathrm{CSloss}}$ is the loss of energy to viscosity of the cell. An additional loss $\left({E}_{\mathrm{lymph}}\right)$ occurs at the lymphangion level due to the behavior of the lymph being pumped

13c $$ \frac{{{\text{dE}}_{{{\text{lymph}}}} }}{{{\text{dt}}}} = \frac{{64\mu L_{v} }}{{\pi D^{3} }}\left( {Q_{1}^{2} + Q_{2}^{2} } \right) $$

At the sliding filament level, energy is lost because not all heads complete the entire powerstroke. Incomplete powerstrokes liberate the same amount of energy but cannot convert all of the energy liberated to work (Barclay & Loiselle 2020; Eisenberg et al. 1980). This is combined with the fact that ATP hydrolysis is irreversible so the excess liberated energy is not re-captured and is lost to entropy as heat (Chapman & Loiselle 2016; Loiselle et al. 2010). The conversion to heat is likely via internal friction between the myosin heads and actin binding sites similar to heat losses in F_0_F_1_-ATPase (Chapman & Loiselle 2016) (Table 2).

**Table 2:** Muscle model parameters other than rate function constants

Symbol	Description	Value [dimension]	Source
$N_{{{\text{Cell}}}}$	Number of LMCs around lymphangion circumference	4 [−]	Unpublished estimate of D. Zawieja
$N_{{{\text{Rows}}}}$	Total number of parallel rows of CEs	3 × 6e3 [−]	Reference value chosen Estimate from SEM image of rat mesenteric lymphatic (Muthuchamy et al. 2003)
${\text{Num}}_{P}$	Number of myosin heads per phasic CE	4500 [−]	Reference value chosen Combined volume estimate from cultured LMCs in (Muthuchamy et al. 2003) with concentration of heads from (Bagshaw 1993)
${\text{Num}}_{T}$	Number of myosin heads per tonic CE	1000 [−]	Reference value chosen Assumed slight reduction in head number from phasic due to less structural organization
$E_{{{\text{Cell}}}}$	Stiffness of LMC cytoskeleton	75 [dyne/cm]	Reference value chosen so that the pressure shape resembles published recordings from rat mesenteric lymphangions In range of (Trepat et al. 2007) for human airway smooth muscle
$\mu_{{{\text{Cell}}}}$	Dynamic viscosity of (one-dimensional) LMC	50 [(dyne s)/cm]	Reference value chosen In range of (Trepat et al. 2007) for human airway smooth muscle
$N_{P}$	Series number of phasic CEs	14 [−]	Made to give approximate lengths of striated CE lengths observed in other muscles
$N_{T}$	Series number of tonic CEs	14 [−]	Made to give approximate lengths of smooth muscle CE lengths observed
$\rho$	Length density of actin binding sites	6e5 [1/cm]	Assumed consistent between CE types. (Huxley 1957) gave an actin site separation of 153 Å for frog sartorius muscle (equivalent to density of 6.5e5[1/cm], 8% difference) Airway smooth muscle (Brook & Jensen 2014)
$h$	Powerstroke length	15.6 [nm]	Assumed consistent between CE types Frog sartorius muscle (Huxley 1957)
$K_{P}$	Stiffness constant of phasic myosin heads	0.4 [dyne/cm]	Frog sartorius muscle (Huxley 1957)
$K_{T}$	Stiffness constant of tonic myosin heads	1.8 [dyne/cm]	Airway smooth muscle (Brook & Jensen 2014)
$c_{{0.5,{\text{Trop}}}}$	Calcium concentration for half-saturation of cardiac TnC	0.27 [μM]	Reference value chosen
$c_{{0.5,{\text{CaM}}}}$	Calcium concentration for half-saturation of CaM	8 [μM]	Reference value chosen
$n_{{m{\text{Trop}}}}$	Hill exponent for CaM calcium saturation	12 [−]	Reference value chosen
$n_{{m{\text{CaM}}}}$	Hill exponent for CaM calcium saturation	1.5 [−]	Reference value chosen
${\text{cycletime}}$	Duration of total contractile cycle	5 [s]	Rat mesenteric lymphatic muscle (Zawieja et al. 1999)
${\text{Ca}}_{{{\text{amp}}}}$	Peak calcium concentration	240 [nM]	Rat mesenteric lymphatic muscle (Zawieja et al. 1999)
${\text{Ca}}_{d}$	Diastolic calcium concentration	140 [nM]	Rat mesenteric lymphatic muscle (Zawieja et al. 1999)
$a_{2}$	Constitutive parameter for intracellular free calcium concentration	24.17 [s^−6^]	Rat mesenteric lymphatic muscle (Zawieja et al. 1999)
$b_{2}$	Constitutive parameter for intracellular free calcium concentration	0.5278 [s^−6^]	Rat mesenteric lymphatic muscle (Zawieja et al. 1999)
$\mu_{T}$	Tonic dashpot (one-dimensional) viscosity constant	10 [(dyne s)/cm]	Reference value chosen
$a$	Constitutive parameter for strain-stiffening of phasic spring	5.1282e−23 [dyne/cm]	Fit to titin (in range from various references) (Kellermayer et al. 2003; Labeit et al. 2003; Linke & Grützner 2008)
$b$	Constitutive parameter for strain-stiffening of phasic spring	7.3838e + 3 [1/cm]	Fit to titin (in range from various references) (Kellermayer et al. 2003; Labeit et al. 2003; Linke & Grützner 2008)

#### Excitation–contraction coupling

To induce contractions, ECC was initiated via a periodic intracellular free calcium concentration $\left(c\right)$. This concentration was used to calculate the saturation of binding proteins. Saturations alter the transition rates between myosin head states to influence force generation (Wang et al. 2008; Wong 1971, 1972; Yochum et al. 2015). The calcium concentration in this model is not affected by the regulatory mechanisms operating in lymphatic muscle (e.g., shear- and pressure-dependent changes in calcium concentration levels and timing).

The rat mesenteric lymphatic vessel calcium measurements of Zawieja and colleagues (Zawieja et al. 1999) were fit, featuring a systolic peak of 232 nM   and diastolic plateau of 140 nM. Calcium oscillations were modelled as sinusoidal oscillations about the diastolic concentration.

14 $$ c\left( t \right) = \left\{ {\begin{array}{*{20}c} {\left( {{\text{Ca}}_{{{\text{amp}}}} - {\text{Ca}}_{d} } \right)\frac{{a_{2} }}{{b_{2} - a_{2} }}\left[ {e^{{ - a_{2} t_{p}^{6} }} - e^{{ - b_{2} t_{p}^{6} }} } \right] + {\text{Ca}}_{d} } & {{\text{if}}\; t_{p} < t_{{{\text{Osc}}}} } \\ {{\text{Ca}}_{d} \left( {1 + {\text{Osc}}_{{{\text{Amp}}}} \sin \left( {\omega_{{{\text{Osc}}}} 2\pi \left( {t_{p} - t_{{{\text{Osc}}}} } \right)} \right)} \right) } & {{\text{if}}\; t_{p} \ge t_{{{\text{Osc}}}} } \\ \end{array} } \right. $$

where ${\mathrm{Ca}}_{\mathrm{amp}}$ is the peak calcium concentration, ${\mathrm{Ca}}_{d}$ is the diastolic calcium concentration, ${a}_{2},{b}_{2}$ are constitutive parameters, ${t}_{p}$ is the time from the beginning of the current cycle, ${t}_{Osc}$ is the time in the current cycle for calcium oscillations onset, ${\mathrm{Osc}}_{\mathrm{Amp}}$ is the amplitude of calcium oscillations as a fraction of the average diastolic calcium, and ${\omega }_{\mathrm{Osc}}$ is the frequency of calcium oscillations.

Calcium oscillations were modelled as sinusoidal fluctuations in diastolic calcium concentration with various amplitudes and frequencies. Amplitudes were expressed as a percentage of the average diastolic calcium concentration. Frequencies were included as the number of oscillations per diastolic period.

The calcium saturations of TnC and CaM were modelled using Hill functions following published models from bladder smooth muscle (Laforêt et al. 2011), uterine smooth muscle (Yochum et al. 2015) and rabbit skeletal muscle (Grabarek et al. 1983). The calcium binding/unbinding rates for both TnC and CaM are much faster than the rates of actomyosin cycling (Cannell & Allen 1984; Faas & Mody 2012; Robertson et al. 1981), so a quasi-steady state can be assumed. Calcium-independent regulatory mechanisms affecting myosin phosphorylation are not considered here.

TnC saturation $\left({S}_{\mathrm{Trop}}\right)$ is calculated using

15a $$ S_{{{\text{Trop}}}} = \frac{{c^{{n_{{m{\text{Trop}}}} }} }}{{c^{{n_{{m{\text{Trop}}}} }} + c_{{0.5,{\text{Trop}}}}^{{n_{{m{\text{Trop}}}} }} }} $$

where ${n}_{m\mathrm{Trop}}$ is the Hill coefficient for TnC, ${c}_{0.5,Trop}$ is the half-saturation concentration for TnC.

CaM saturation $\left({S}_{CaM}\right)$ is calculated using

15b $$ S_{{{\text{CaM}}}} = \frac{{c^{{n_{{m{\text{CaM}}}} }} }}{{c^{{n_{{m{\text{CaM}}}} }} + c_{{0.5,{\text{CaM}}}}^{{n_{{m{\text{CaM}}}} }} }} $$

where ${n}_{m\mathrm{CaM}}$ is the Hill coefficient for CaM, and ${c}_{0.5,\mathrm{CaM}}$ is the calcium concentration at which half of the CaM calcium binding sites are occupied, representing the affinity of the protein to calcium.

The phosphorylation and attachment rates were multiplied by the saturation of the regulatory proteins because the over all saturation is representative of the probability that any given protein will be saturated. The probability of state transition is then the product of the probability that the myosin site is “active” (regulatory protein saturated) and the probability of transition given that a site is “active.”

16a $$ k_{1} = S_{{{\text{CaM}}}} K_{1} $$

16b $$ k_{6} = S_{{{\text{CaM}}}} K_{6} $$

16c $$ f = \left\{ {\begin{array}{*{20}l} 0 & \,{{\text{if}} \;x < 0} \\ {S_{{{\text{Trop}}}} f_{1} x/h} & {{\text{if}}\; 0 \le x \le h} \\ 0 & {{\text{if}}\; h < x} \\ \end{array} } \right. $$

where ${K}_{1}={K}_{6}$ is the maximum phosphorylation rate and ${f}_{1}$ is the rate constant for attachment of phasic myosin heads (Table 3).

**Table 3:** Over all sensitivity measures for each parameter

Variable	$c_{{0.5,{\text{Trop}}}}$	${\text{Ca}}_{{{\text{amp}}}}$	$n_{{m{\text{Trop}}}}$	$g_{2}$	$N_{{{\text{Cell}}}}$
Average PRCC	0.251	0.489	0.166	0.130	0.234

### Lumped parameter model of lymphatic pumping

To test the response of the muscle model, it was incorporated into an existing one-dimensional ordinary differential equation-based lumped parameter model of lymphangion pumping that has been extensively validated (Bertram et al. 2017; Bertram, et al., 2014b, a; Bertram et al., 2011; Bertram et al. 2014b; Bertram et al. 2016; Jamalian et al. 2013; Jamalian et al. 2016; Jamalian et al. 2017).

The model includes conservation of mass, conservation of axial momentum and a circumferential wall-force balance into which the intrinsic contractions from the above cell contractions were incorporated. Momentum conservation includes terms for resistances of the lymphangion body and valves. Pressure boundary conditions are applied at the inlet and outlet of a single lymphangion or chain of lymphangions. In the results presented here, a single lymphangion is modelled. The wall-force balance is based on the central lymphangion pressure $\left({p}_{m}\right)$ and includes a passive elasticity term (pressure-diameter tube law fitted to experimental data), intrinsic contractions, and the pressure external to the lymphangion. The axial length of the lymphangion was assumed not to vary over the course of a contractile cycle in line with previous iterations of the lymphangion model. In previous iterations of this lymphangion model, the intrinsic contractions were based on prescribed diameter- and time-dependencies. This work replaces the applied intrinsic contractions with the lymphatic muscle model. Passive viscoelasticity of LMCs was included in addition to the passive pressure-diameter fit because the passive, purely elastic tube law is dominated by collagen and elastin fibers in the vessel wall rather than cytoskeletal stiffness of the LMCs.

Several LMCs in a single layer around the lymphangion are assumed to be circumferentially oriented. Direct transmission of force between LMCs is assumed (no viscous losses or elastic storage in the extracellular matrix).

The length of each LMC $\left({L}_{\mathrm{Cell}}\right)$ is assumed to be the same for all cells in a single layer surrounding a lymphangion. Cell length is calculated from a circumferential length balance.

17 $$ L_{{{\text{Cell}}}} = \frac{\pi D}{{N_{{{\text{Cell}}}} }} $$

The wall-force balance with zero intrinsic force is

18a $$ P_{m} - P_{{{\text{ext}}}} - \frac{{2E_{{{\text{Cell}}}} L}}{DL} = f_{{{\text{pas}}}} \left( {D_{0} } \right) $$

where ${D}_{0}$ is the passive diameter (zero intrinsic force) under the prescribed pressure conditions.

The reference length (for zero force) of the phasic parallel spring is therefore set to be the phasic CE length with zero intrinsic force and multiplied by a reducing factor of 0.1 to ensure contractile force giving a reference phasic spring length of

18b $$ Y_{{P,{\text{ref}}}} = 0.1\frac{{\pi D_{0} }}{{N_{{{\text{Cell}}}} \left( {N_{P} + N_{T} } \right)}} $$

The ejection fraction (*EF *), a commonly used metric for lymphatic pumping, was calculated from the minimum and maximum diameters to compare the simulation pumping with published experiments.

19 $$ EF = 100\frac{{\max \left( D \right)^{2} - \min \left( D \right)^{2} }}{{\max \left( D \right)^{2} }} $$

The useful energy transferred to fluid movement was calculated from the volumetric outflow rate and the adverse pressure difference following (Bertram et al. 2016)

20 $$ \frac{{{\text{dE}}_{{{\text{fluid}}}} }}{{{\text{dt}}}} = Q_{2} \left( {P_{b} - P_{a} } \right) $$

### Solution method

The solution to this system of equations was obtained in two stages. In the first stage, the resting (zero muscle force) equilibrium diameter was calculated based on the mid-lymphangion pressure. This was followed by solution of the full coupled model of lymphangion and muscle (flowchart in the Supplementary material). The initial conditions for the lymphangion were diameter $\left(0.02 \mathrm{cm}\right)$, upstream pressure $\left(={p}_{a}\right)$ and downstream pressure $\left(={p}_{b}\right)$. Equilibrium diameter was obtained using the adaptive time step MATLAB solver ODE15S. Phasic and tonic myosin heads were initialized as all detached, and tonic myosin heads all dephosphorylated. CEs were initialized to equal lengths. The reference lengths for the spring in parallel with phasic CEs and LMC stiffness were calculated as fractions of the initial length. This was done to ensure that the phasic parallel spring and the LMC stiffness contributed contractile forces. A second solver was then used to develop periodic contractions with the coupled lymphangion and muscle equations. The ordinary differential equation for diameter was solved using a first-order forward finite difference. The partial differential equations for myosin head states of both isoforms were solved using a second-order Godunov solver (Brook et al. 1999; Brook & Jensen 2014; Hiorns et al. 2014) (see Supplementary material). This solver is an explicit upwind finite volume scheme. A first-order half-time step is calculated with the effects of head transitions neglected in calculating the convective fluxes. This uses Riemann solutions at the boundaries of each displacement cell, and a separate source/sink term is used for transitions. The head distributions after this first half-step are used to calculate updated fluxes through a spatial gradient for calculation of a full time step. The time step was determined using a Courant–Friedrichs–Lewy condition $\left(\mathrm{dt}=0.8\mathrm{dx}/\mathrm{max}\left(\mathrm{abs}\left[{v}_{P},{v}_{T}\right]\right)\right)$ and a maximum step size of ${10}^{-4} \mathrm{s}$. The maximum step size was limited to ensure that during diastole when the velocities are small the model was still stable and could resolve the diameter and pressure. Some instabilities in model results were initially observed, and this was identified to be due to the inclusion of ${\mu }_{\mathrm{Cell}}$. Decreasing the time step removed these instabilities. Displacement integrals were evaluated using trapezoidal integration. Algebraic equations for lymphangion pressures were solved at each time step using the force resulting from updated head states to give updated pressures used in the following time step. Simulations were considered periodic when the following conditions were met:

21a $$ 100\left| {\frac{{\overline{Q}_{2} - \overline{Q}_{1} }}{{\overline{Q}_{2} }}} \right| < 1 $$

21b $$ \left| {\overline{{\frac{{{\text{dY}}_{T} }}{{{\text{dt}}}}}} } \right|{\text{cycletime}} < 10^{ - 2} {\text{cm}} $$

21c $$ 100\left| {\frac{{\overline{{F_{T} }} - \overline{{F_{T} }}_{{\Pr {\text{ev}}}} }}{{\overline{{F_{T} }} }}} \right| < 2 $$

where ${\overline{{F }_{T}}}_{\mathrm{Prev}}$ is the average of tonic CE force over the previous cycle.

Refer to the Supplementary material for details on verification of periodicity conditions under reference conditions. The simulation under reference conditions took 6.7 h on a laptop with an AMD Ryzen 7 3700U processor (quad core 2.3 GHz per core) and 8 GB of RAM. Periodicity was reached after 10 contractile cycles. With the use of different input values, the duration of simulations varied in the range of approximately 4–10 h (9–16 contractile cycles). Simulations struggled to reach these periodicity conditions when the lymphangion was chronically constricted, so simulations were run for a maximum of 10 cycles. For the simulations run for 10 cycles, the magnitude difference in valve flows was < 0.09 mL/hr , change in tonic CE length during the final cycle was < 2.5 × 10^–6^ µm and the difference in average tonic force between the final two cycles was < 2.3%. These values represent minor changes between contractile cycles, relative to the magnitude of cyclic variations, that do not affect over all conclusions from the model.

## Parameter sensitivity analysis

This sensitivity analysis focusses on the parameters of the muscle model only, without the parameters involved in the lymphangion model. Our model has many independent parameters ($\varphi =32$), and the runtime is significant (7 hours under reference conditions). For the full set of parameters, a Latin hypercube sensitivity analysis with 100 trials would take approximately 30,800 days to run without any parallelisation. This time scales with ${\varphi }^{\varphi +1}$ so screening was used to reduce the dimensionality of the parameter sensitivity analysis and make it more practical.

The most commonly used screening method in engineering applications is one-at-a-time sensitivity analysis (Iooss & Lemaître 2015). We therefore ran an initial screening sensitivity analysis by individually varying parameters from their reference value and examining the effect on the average outflow. The five parameters with the greatest effect on the average flow as defined by the ratio of normalized change in outflow to normalized change in parameter value (Eq. 22) were considered the most sensitive, and a more detailed general sensitivity analysis was run with this reduced set of parameters.

### One-at-a-time analysis

One-at-a-time is a local sensitivity analysis relating model outputs for altered parameter values to results from the reference condition. The pressure conditions were maintained at ${p}_{a}=3 {\mathrm{cmH}}_{2}\mathrm{O}$, ${p}_{b}=3.1 {\mathrm{cmH}}_{2}\mathrm{O}$ and ${p}_{\mathrm{ext}}=2 {\mathrm{cmH}}_{2}\mathrm{O}$. Sensitivity of the results to variations in the parameter were assessed using the ratio of the normalized change in average flow to the normalized change in input.

22 $$ S_{i,j} = \frac{{\left( {\overline{Q}_{{{\text{ref}}}} - \overline{Q}_{i,j} } \right)/\overline{Q}_{{{\text{ref}}}} }}{{\left( {{\text{Parameter}}_{{{\text{ref}},j}} - {\text{Parameter}}_{i,j} } \right)/{\text{Parameter}}_{{{\text{ref}},j}} }} $$

where *i* indexes the adjusted parameter values, *j* indexes the parameter, ${\overline{Q} }_{\mathrm{ref}}$ is the average outflow with reference parameter values, ${\overline{Q} }_{i,j}$ is the average outflow with the adjusted parameter value, ${Parameter}_{\mathrm{ref},j}$ is the reference value of the parameter and ${Parameter}_{i,j}$ is the adjusted parameter value.

This sensitivity index can be positive (increase in average flow with increase in parameter) or negative (decrease in average flow with increase in parameter) and an absolute value of one represents a linear increase, absolute value greater than one represents a superlinear relationship and less than one a sublinear relationship. See the Supplementary material for a table of results obtained from this analysis.

Designating parameters with a maximum $\left|S\right|>1.2$ as sensitive yielded five parameters for a more in-depth global sensitivity analysis using Latin hypercube sampling. These were the peak calcium, the phasic calcium binding properties, the phasic detachment rate for negative displacements, and the series number of LMCs around the lymphangion.

### Latin hypercube sensitivity analysis

The range of possible values for the *k*=5 parameters were each divided into $\psi =k+1=6$ regions as required by Latin hypercube sampling (Marino et al. 2008). We assumed a uniform probability distribution for the range of values of each parameter. The Latin hypercube sampling method randomly takes values from each parameter’s probability distribution (one value from each region) without repetition to ensure that the entire range of values for each parameter is sampled. These random values for each parameter are then assembled into a matrix with $\psi$ rows for the number of samples (equivalently model runs) and *k* columns. One matrix represents a single Latin hypercube trial that provides a single measure of the correlation between the parameters and the average outflow. Each trial therefore involved six model calls and resulted in six sample values of average flow. Multiple trials are then required to allow for statistical separation of the influence of each parameter as they are being simultaneously randomly sampled so we ran 100 trials.

Sensitivity of the model outflow on each of the parameters was calculated using the partial rank correlation coefficient (PRCC). The PRCC measures the linear relationship between the input and the output, varying between − 1 and 1. Negative values mean that the output decreases with increasing input and positive increasing with increasing output and larger magnitudes indicating stronger relationships. One PRCC value was calculated for each trial using the 6 samples. These correlation coefficients were then plotted using box plots, and an over all sensitivity measure was calculated from their mean over all trials.

The equation for the PRCC for a single trial is

23a $$ PRCC_{j} = \frac{{{\text{Co}}{\text{var}} {\text{iance}}}}{{\sqrt {{\text{Variance}}\left( {X_{j} } \right){\text{Variance}}\left( Y \right)} }}\quad j = 1, \ldots ,k $$

23b $$ {\text{Co}}{\text{var}} {\text{iance}} = \frac{{\mathop \sum \nolimits_{i = 1}^{\psi } \left( {X_{ij} - \overline{X}} \right)\left( {Y_{i} - \overline{Y}} \right)}}{\psi - 1} $$

where $X$ is input (overbar is averaged over the $\psi$ samples), $Y$ is output (overbar is averaged over the $n$ samples), $i$ indexes the sample (combination of randomized parameter values for a single model run), and $j$ indexes input parameter

23c $$ {\text{Variance}}\left( {X_{j} } \right) = \frac{{\mathop \sum \nolimits_{i = 1}^{\psi } \left( {X_{ij} - \overline{X}} \right)^{2} }}{\psi - 1} $$

**Fig. 3:**
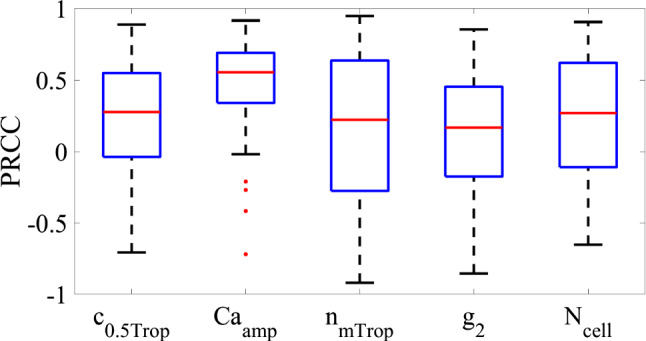
Boxplots of the PRCC for each parameter showing the variation between trials

This analysis showed that the model was most sensitive to the peak calcium concentration (PRCC value of 0.489) with the next two sensitive parameters of ${c}_{0.5,\mathrm{Trop}}$ and ${N}_{\mathrm{Cell}}$ showing PRCC values approximately half of that for the peak calcium concentration (0.251 and 0.234, respectively) (Fig. 3).

## Results

### Model performance under reference conditions

**Fig. 4:**
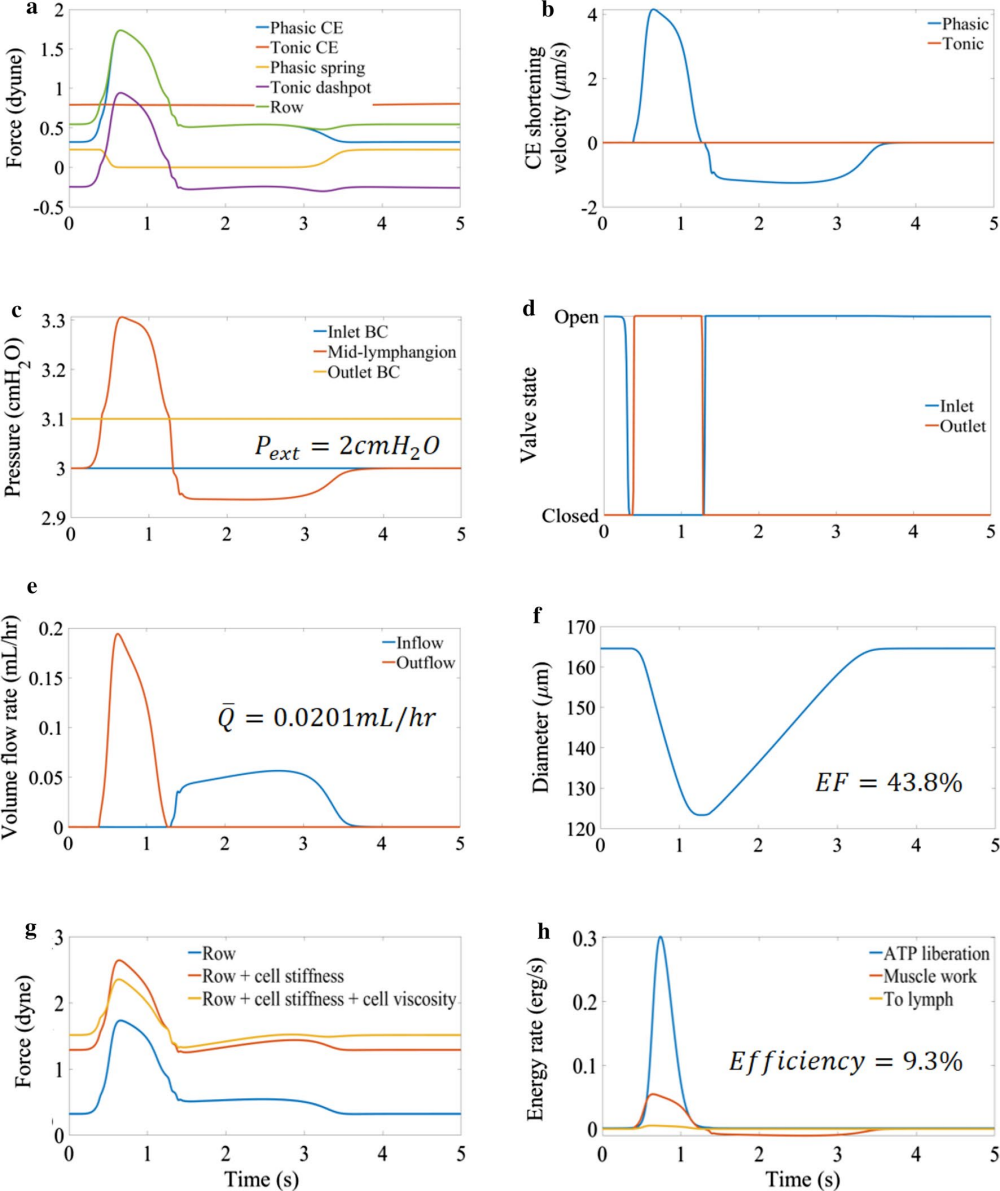
Panel of plots summarizing results for a single lymphangion contractile cycle under reference conditions (${\text{P}}_{{\text{a}}} = 3.0{\text{ cmH}}_{2} {\text{O}}$, ${\text{P}}_{{\text{b}}} = 3.1{\text{ cmH}}_{2} {\text{O}}$ and ${\text{P}}_{{{\text{ext}}}} = 2.0{\text{ cmH}}_{2} {\text{O}}$). **a** summarises the force contributions from subcellular components (CEs, phasic spring and tonic dashpot) **b** is the shortening velocity of CEs showing the greatly reduced tonic CE velocity resulting from the presence of the tonic dashpot **c** is the pressure in the lymphangion compared to boundary conditions showing the increase to expel fluid and the decrease to refill the lymphangion **d** shows the opening and closing of the valves **e** shows the volume flow rates **f** shows the diameter of the lymphangion **g** shows the contributions of the cell viscoelasticity and CEs to the over all force generated by an LMC **h** shows the rates of useful work done by the muscle compared to the energy liberated by ATP hydrolysis to model the metabolic efficiency of lymphatic muscle and the useful energy imparted to the fluid

Plots of the CE row forces (note that these are the total values for the lymphangion) against time show that tonic force is almost constant throughout the contractile cycle with a range of 2.7% the average value under reference conditions (Fig. 4a). The small variation in tonic force is due to the resistance of the tonic dashpot to any changes in tonic CE length. In the absence of a tonic dashpot, the phasic CEs would cause too much convection for the tonic CEs to form sufficient cross-bridges for physiologic force generation. The phasic spring provides a means for the tonic CE force to be transmitted over the whole cell when the phasic CE force drops in diastole, thus setting the diastolic diameter. The phasic force (Fig. 4a) increases by $1.4\, \mathrm{dyne}$ in response to increased calcium concentration to cause the lymphangion to propel fluid downstream, resulting in a peak cell force of approximately $2.4\, \mathrm{dyne}$ (Fig. 4g). The mid-lymphangion pressure increased to a peak value of $0.21\, {\mathrm{cmH}}_{2}\mathrm{O}$ above the outlet (Fig. 4c) and reduced to $0.06\, {\mathrm{cmH}}_{2}\mathrm{O}$ below the inlet pressure to create a suction effect for diastolic filling (Fig. 4c). The time-averaged flow rate was $0.02\, \mathrm{mL}/\mathrm{hr}$ with a peak of $0.200\, \mathrm{mL}/\mathrm{hr}$ (Fig. 4e), resulting from a decrease in diameter of 41 µm below the diastolic value of 165 µm for an ejection fraction of 44% (Fig. 4f). Muscle cells converted approximately 9.3% of free energy from ATP to work. The peak rate of energy liberation by the lymphangion was $0.30\, \mathrm{erg}/\mathrm{s}$ and peak rate of muscle work was $0.055\, \mathrm{erg}/\mathrm{s}$ and, during diameter recovery, the inflowing lymph performed work lengthening the muscle with a peak value of $0.011\, \mathrm{erg}/\mathrm{s}$ (Fig. 4h). Useful output work for fluid motion reached a peak value of $0.005\, \mathrm{erg}/\mathrm{s}$ and 30.3% of the muscle work was transferred to the fluid (corresponding to 2.8% of energy liberated).

### Comparison to experimental data

**Fig. 5:**
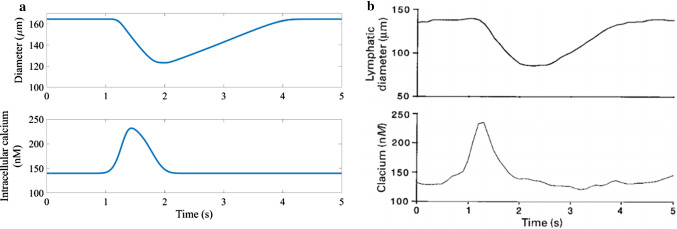
Comparing the diameter results of the muscle model at a transmural pressure $1{\text{ cmH}}_{2} {\text{O}}$ and no axial pressure difference to experimental data obtained from rat mesenteric lymphatics. **a** diameter and calcium from the coupled muscle-lymphangion model **b** diameter and calcium tracings from the experimental results of (Zawieja et al. 1999)

Model results under conditions of transmural pressure 1 cmH_2_O and constant axial pressure (${p}_{a}={p}_{b}$) were primarily compared to the results of Zawieja (Zawieja et al. 1999) obtained from cannulated rat mesenteric lymphatics. The calcium input for the model was a fit of the calcium tracing of Zawieja’s results, allowing for closer comparison of the results (Fig. 5). The shape of the diameter-time plot was similar to that recorded by Zawieja, including the phase shift relative to the calcium transient, and the ejection fraction was within 1% of the results obtained by Zawieja at $1\, {\mathrm{cmH}}_{2}\mathrm{O}$ (46% from model, 45% in experiments). Normalizing the diameter values to the diastolic value showed that the muscle model generated smaller changes than recorded in Zawieja’s experiments (normalized contracted diameter 0.73 compared to 0.60).

**Fig. 6:**
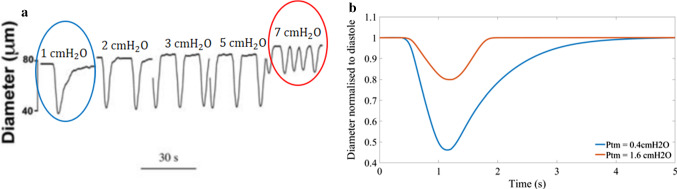
Comparing the diameter-time shape for lymphangions recorded experimentally to coupled muscle-lymphangion model results **a** diameter of cannulated rat mesenteric lymphatics at various transmural pressures (1,2,3,5,7 ${\text{cmH}}_{2} {\text{O}}$, increasing in 30 s intervals) (Zawieja 2009) showing that the linear recovery of diameter occurs at higher transmural pressures **b** results from the muscle-lymphangion model transmural pressures of 0.4 ${\text{cmH}}_{2} {\text{O}}$ showing the exponential shape of diameter-time during recovery and 1.6 ${\text{cmH}}_{2} {\text{O}}$ showing the more linear diameter-time recovery shape

In a series of experiments, (Zawieja 2009) increased the transmural pressure applied to cannulated rat mesenteric lymphatics (1, 2, 3, 5, 7 ${\text{cm}} \, {\text{H}}_{2}{\text{O}}$) every 30 s the diameter-time relationship during recovery appeared exponential at lower transmural pressures (1 ${\text{cm}} \, {\text{H}}_{2}{\text{O}}$) and more linear after increasing the transmural pressure (2 ${\text{cm}} \, {\text{H}}_{2}{\text{O}}$ and higher) as shown in Fig. 6a. The reported ejection fraction for the transmural pressure results at 1 ${\text{cm}} \, {\text{H}}_{2}{\text{O}}$ in this set of experiments was higher than the previous experimental results and the model results at 65%. At decreased transmural pressure (0.4 ${\text{cm}} \, {\text{H}}_{2}{\text{O}}$), the model exhibited a more gradual diameter-time shape during recovery (Fig. 6b).

### Effects of important parameters on pumping performance

**Fig. 7:**
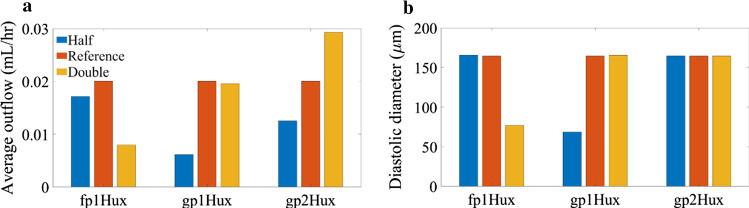
Sensitivity of the model to phasic rates **a** rate of fluid output **b** diastolic size of the lymphangion. When the attachment is too high (${\text{f}}_{1}$ double) or the detachment is too low (${\text{g}}_{1}$ half), the phasic force during diastole causes constriction of the diameter, so there is greatly reduced flow

The average outflow was more sensitive to ${f}_{1}$ than ${g}_{1}$, decreasing by 15% with halving ${f}_{1}$ and by 2% with doubling ${g}_{1}$. Outflow decreased by approximately 60% when ${f}_{1}$ was doubled and 69% when ${g}_{1}$ halved. This was due to the increased maintenance of cross-bridges causing continuous constriction of the lymphangion (Fig. 7a,b). The average flow increased by 46% when ${g}_{2}$ was doubled and decreased by 38% when ${g}_{2}$ was halved (Fig. 7a).

**Fig. 8:**
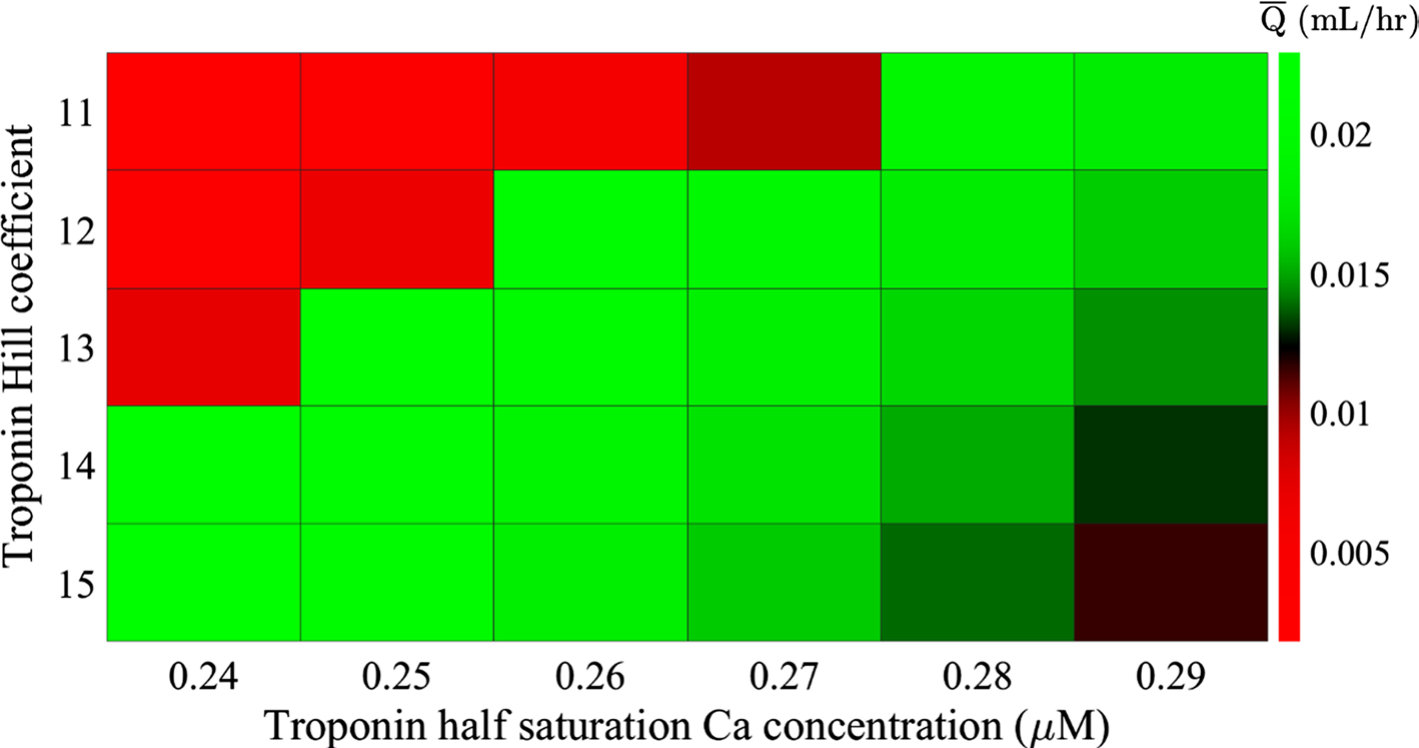
Sensitivity of outflow to variations in the calcium binding properties of TnC. There is a combination of TnC Hill coefficients that result in effective pumping

There was an optimal range of combinations of troponin Hill coefficient and calcium saturation for half-saturation that generated a high average flow (Fig. 8). This optimal range of ${n}_{m\mathrm{Trop}}$, ${c}_{0.5,\mathrm{Trop}}$ combinations occurred where the peak concentration is large (between 4.2 and 38.1%) with a low enough diastolic saturation (in the range of 0.003 to 0.06%) to prevent constant constriction. Constant constriction to diameters < 86 µm occurred when both ${n}_{m\mathrm{Trop}}$ and ${c}_{0.5,\mathrm{Trop}}$ were low. This was due to an increased diastolic saturation (0.07%) causing an increased diastolic force (phasic CE force $> 0.59\, {\text{dyne}}$). High combinations of $n_{{m{\text{Trop}}}}$, $c_{{0.5,{\text{Trop}}}}$ caused a low peak saturation and small phasic contraction amplitudes so the lymphangion was unable to generate significant flow. Neglecting constricted results, the average outflow decreased with increasing half-saturation concentration and this decrease in flow was because of a decreasing peak saturation and peak phasic CE force (see Table 4). Varying $c_{{0.5,{\text{CaM}}}}$ between 4  µm and 20 µm caused variations in the average flow of < 1%.

**Table 4:** Decreases in average flow with increasing half-saturation concentration

$n_{{m{\text{Trop}}}}$	Decrease in $\overline{Q} \left[ {mL/{\text{hr}}} \right]$	Decrease in peak $S_{{{\text{Trop}}}} \left[ \% \right]$	Decrease in peak $F_{P} \left[ {{\text{dyne}}} \right]$
11	0.0017	3.29	0.027
12	0.0052	13.8	0.082
13	0.0075	22.1	0.122
14	0.0099	33.9	0.166
15	0.0105	34.0	0.197

### Pressure-dependent effects

**Fig. 9:**
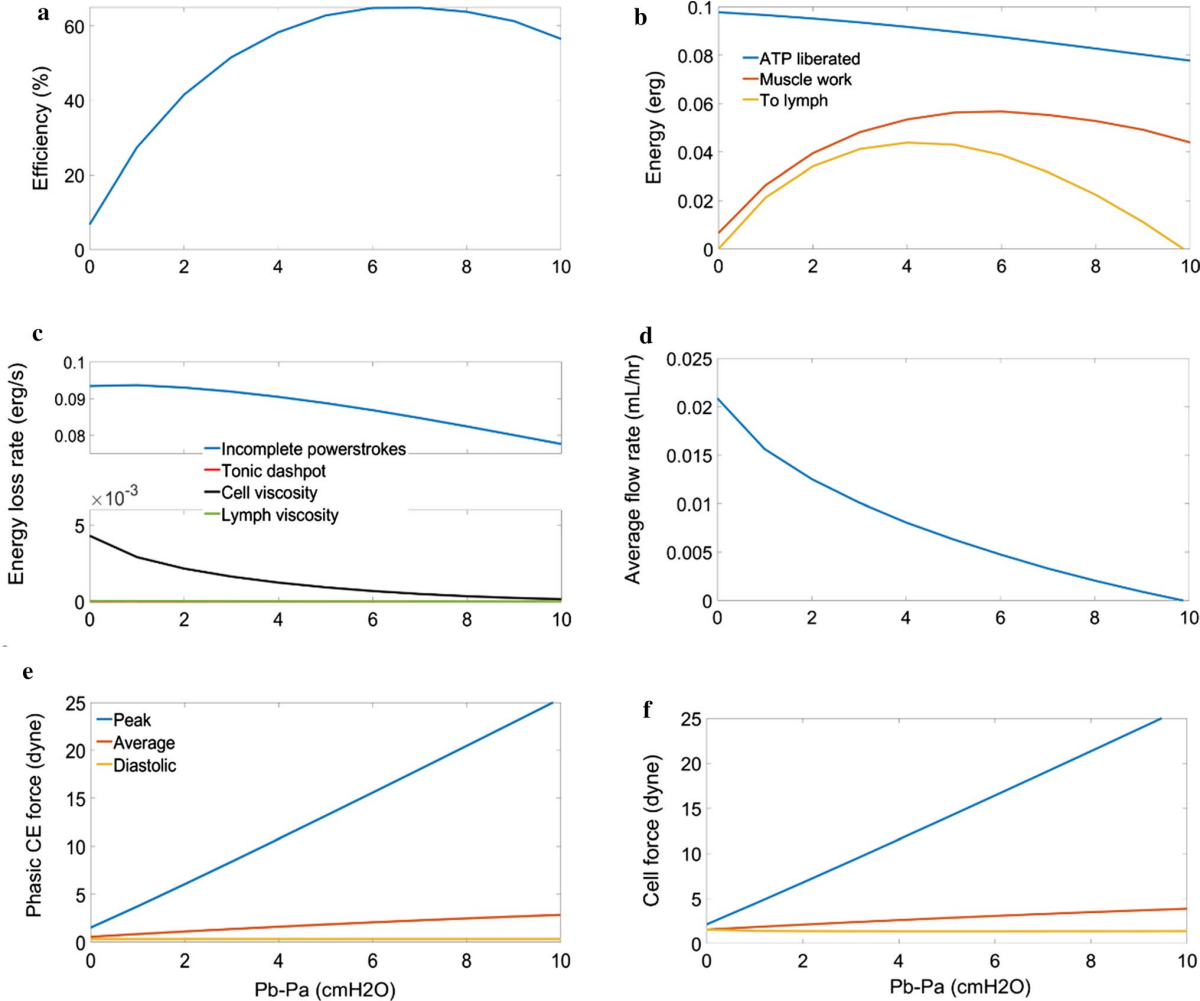
Panel of plots summarizing the effect of increasing the outlet pressure for consistent inlet ($3{\text{ cmH}}_{2} {\text{O}}$) and external pressures ($2{\text{ cmH}}_{2} {\text{O}}$). **a** shows an increase in efficiency with increased afterload until a point after which there is a slight decrease until pump failure (defined as zero net outflow) **b** compares the energy liberated to the work done by the muscle and the useful work done on the lymph **c** shows that the viscous losses were much lower than losses to incomplete powerstrokes **d** shows the decrease in outflow from the increased load **e** shows the response of increasing phasic force to increasing load **f** shows that the increasing phasic force causes an increasing cell force

The efficiency of LMCs increases with increasing adverse pressure difference to a peak value of 65% at $7\, {\text{cmH}}_{2} {\text{O}}$ (Fig. 9a) before decreasing to 56.5% at $10\, {\text{cmH}}_{2} {\text{O}}$. Energy losses to viscosity are much lower than to inhomogeneities in cross-bridge attachment/detachment displacements (Fig. 9c). The efficiency increases with $p_{b}$ because there is reduced outflow (Fig. 9d), meaning that there is less convection and more heads can complete the powerstroke. Comparing the muscle work and fluid pumping work shows a similar form for both with a peak cell work of $0.0566\, {\text{erg}}$ at $6\, {\text{cmH}}_{2} {\text{O}}$ and a peak fluid work of $0.0440\, {\text{erg}}$ at $4\, {\text{cmH}}_{2} {\text{O}}$(Fig. 9b). This, combined with the decreasing energy liberation, resulted in a peak of 86% work transfer from muscle to fluid at $2\, {\text{cmH}}_{2} {\text{O}}$ and 48% efficiency of energy liberated to fluid work at $5\, {\text{cmH}}_{2} {\text{O}}$ (Fig. 9b). The decreased convection also increases the peak force generation by phasic CEs (Fig. 9e) from $1.52\, {\text{dyne}}$ at an adverse pressure difference of $0\, {\text{cmH}}_{2} {\text{O}}$ to $25.40\, {\text{dyne}}$ at 10 ${\text{cmH}}_{2} {\text{O}}$. The increased phasic force caused an increase in the peak cell force (Fig. 9f) from $2.13\, {\text{dyne}}$ at an adverse pressure difference of $0\, {\text{cmH}}_{2} {\text{O}}$ to $26.32\, {\text{dyne}}$ at 10 ${\text{cmH}}_{2} {\text{O}}$. The force increase was linear because the outlet pressure was increased linearly, and the maximum mid-lymphangion pressure barely increased above the outlet pressure in each case, meaning that the required force increase was linear. A maximum flow of $0.021\, {\text{mL}}/{\text{hr}}$ was achieved when the outlet pressure was the same as inlet pressure (Fig. 9d) and pumping had failed (no net outflow) at $10\, {\text{cmH}}_{2} {\text{O}}$.

**Fig. 10:**
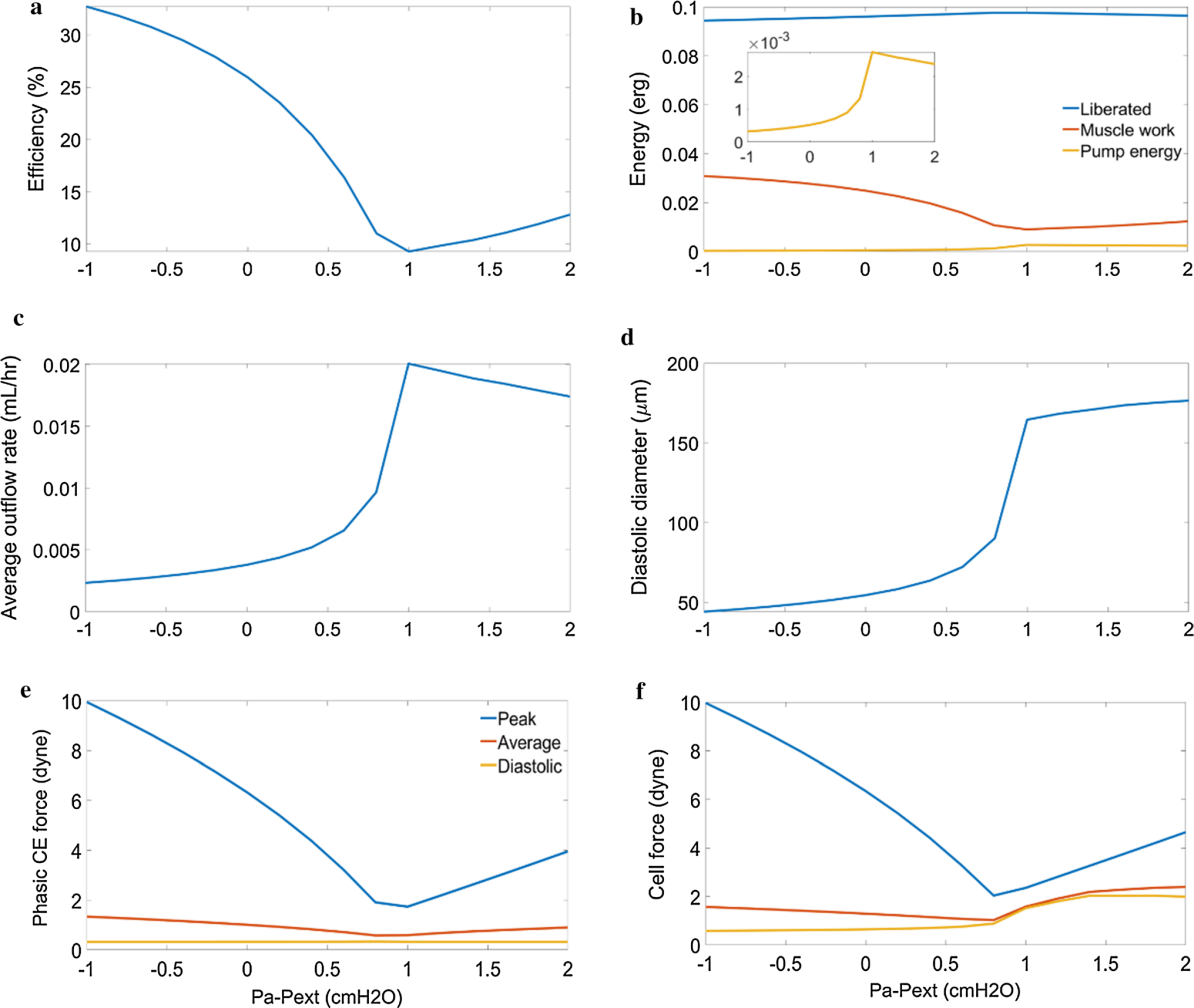
Panel of plots summarizing the effect of simultaneously increasing inlet and outlet pressure for constant external pressure ($2{\text{ cmH}}_{2} {\text{O}}$). **a** shows the efficiency of the muscle which follows an inverse relation to the average outflow **b** shows compares the energy liberated from ATP to the work done by the muscle and energy imparted to the fluid **c** shows the outflow change in response to varying transmural pressure with a peak of ${ }0.0256{\text{ mL}}/{\text{hr}}$ at a transmural pressure of $1{\text{ cmH}}_{2} {\text{O}}$ **d** shows the collapse of lymphangions with transmural pressure lower than $1{\text{ cmH}}_{2} {\text{O}}$, reducing the stroke volume available for pumping **e** shows variations in phasic force which was much greater in collapsed lymphangions **f** shows that the cell force follows phasic CE force

Varying the transmural pressure by simultaneously changing $p_{a}$ and $p_{b}$ showed that the efficiency of lymphatic muscle followed an inverse relation to average flow (Fig. 10a,c) with collapse at low transmural pressures (0.8 ${\text{cmH}}_{2} {\text{O}}$ and lower) causing low stroke volume ($< 14\, {\text{nL}}$ compared to $28\, {\text{nL}}$ at $1\, {\text{cmH}}_{2} {\text{O}}$) (Fig. 10c,d). This was because of the reduced velocity resulting in lower myosin turnover (Fig. 10d) as in the adverse pressure difference results. The fluid work follows average outflow because the adverse pressure difference was constant and had a peak value of $0.003\, {\text{erg}}$ at a transmural pressure of $1\, {\text{cmH}}_{2} {\text{O}}$ (Fig. 10b,c). There was an optimal transmural pressure for greatest average outflow of $0.020\, {\text{mL}}/{\text{hr}}$ (corresponding to minimum efficiency of 9.3%), which occurred during the reference conditions (Fig. 10a). At transmural pressures greater than the optimal, there is increased force required to contract (Fig. 10e,f), reducing the flow similarly to increasing the outlet pressure (Fig. 10c).

### Additional pumping due to calcium transients

**Fig. 11:**
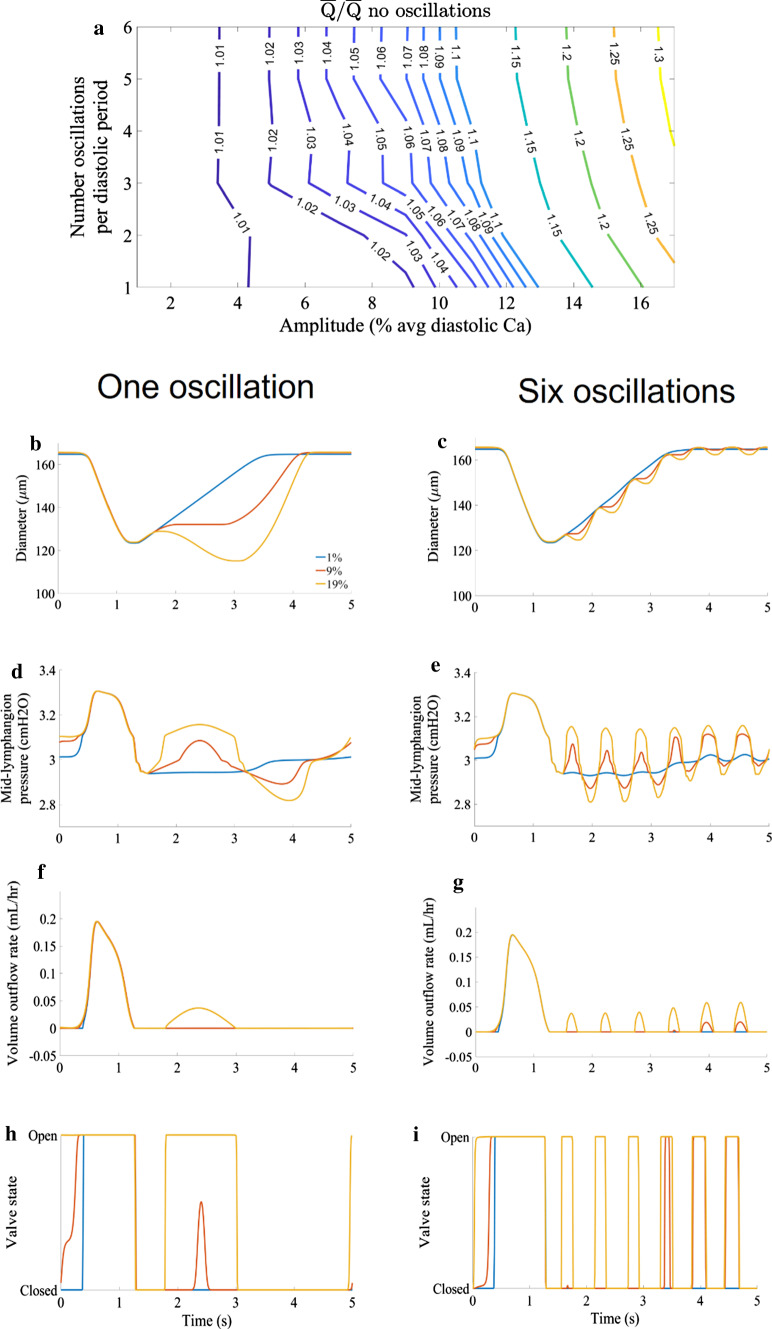
Panel of plots showing the effects of varying both amplitude and frequency of calcium oscillations with all combinations of amplitude and frequency increasing flow relative to the case without calcium oscillations. **a** contours of average outflow normalized to the results without calcium oscillations showing that higher flow is obtained at lower amplitudes when the frequency is increased. **b,c** variation in time-dependence of diameter **d,e** variation in time-dependent mid-lymphangion pressure **f,g** variation in time-dependence of outflow **h,i** variation in time-dependence of outlet valve resistance, showing the opening and closing with calcium oscillations

Including calcium oscillations increased the average flow rate (Fig. 11a) with one oscillation at 1% amplitude causing only about a 0.3% increase. The oscillations without complete opening/closing of the outlet valve were still able to increase outflow because they opened the valve slightly. Oscillation peaks of course resulted in decreases in diameter (Fig. 11b,c) with the greatest occurring at and 19% amplitude. The diameter decreases were due to an increase in pressure (Fig. 11d,e) as a result of the increased calcium allowing more heads to bind. There were low amplitude (compared to action potential, maximum peak was approximately 18.9% action potential peak) peaks in outflow with oscillatory increases in pressure from oscillations (Fig. 11f,g). Valves start rapidly opening and closing at lower amplitudes when the frequency is greater (Fig. 11h,i). The increase in flow became less sensitive to amplitude when the valves started to fully open and close with each depolarization.

## Discussion and conclusions

We have developed a computer model of the subcellular mechanisms of lymphatic muscle contraction that, on coupling with a well-characterized macroscale model of lymphangion pumping, produces flow, diameter, and pressure traces similar to those from experiments on rat mesenteric lymphatics. This model could be used with adjusted parameter values to model other lymphatics with phasic contractions. Ejection fraction and average outflow under reference conditions are within the range previously reported from experiments (see, for example, the published ranges in (Davis et al. 2012) though note the different pressure conditions). The particular arrangement of CEs with the phasic spring and tonic dashpot was the only one that produced physiologic results, out of the many we tried. In terms of conceptual ties to anatomical features, the phasic spring could represent the elasticity of titin while the tonic dashpot could represent the effects of a combination of tonic contractile machinery and the fluid environment around the smooth muscle components. Smooth muscle contains a protein similar to titin, referred to as smitin (Chi 2007; Chi et al. 2005; Kim & Keller III 2002). Both titin and smitin can attach to smooth and striated actin and myosin (Chi 2007; Chi et al. 2005; Kim & Keller III 2002). This implies that titin, smitin, or some combination thereof can provide a direct connection between phasic and tonic CEs in LMCs. No studies have investigated the potential presence of titin or smitin in LMCs. Quantitative assays for distributions of titin and/or smitin in LMC cells would provide additional clarity on the subcellular contractile element structure. In the current version of the model, cell stiffness and viscosity are included as parallel passive elements, meaning that the response of the LMC to instantaneous force is viscoelastic. Varying $E_{{{\text{Cell}}}}$ and $\mu_{{{\text{Cell}}}}$, however, showed that $\mu_{{{\text{Cell}}}}$ had little effect on the model response, meaning that the response is dominated by elastic effects. Understanding the molecular functioning of LMCs is a step toward more physiologic modelling of lymphatic function that is necessary for greater understanding of the system’s performance (Margaris & Black 2012) and is useful for identifying potential pharmaceutical interventions. Our model (and indeed all models of active lymphatic pumping) would benefit greatly from the ability to culture LMCs while maintaining the contractile phenotype, as it would facilitate the exploration of how these cells adapt their contractility in response to physical or biochemical cues.

Our model provides the first estimates of the energetics and efficiency of individual LMCs, and their dependence on lymphangion upstream, downstream, and external pressures. There are, unfortunately, no experimental data with which to compare the energy conversion predictions of the model, so the energetics are included here as estimates that will hopefully be comparable with experimental estimations of ATP usage often performed by measuring the concentration of inorganic phosphate through, for example, fluorescent protein MDCC-PBP (He et al. 2000) and heat generation. The model calculates the efficiency of cross-bridge cycling converting chemical energy of ATP to useful work, ignoring the efficiency associated with the metabolism of storing energy in ATP. The thermodynamic cycling efficiency was calculated from the ratio of work done to free energy from ATP hydrolysis. Another commonly used measure of cross-bridge efficiency is mechanical, based on the ratio of work done to enthalpy (free energy + entropy × temperature). The thermodynamic efficiency estimates from our model are within the ranges of published values for other muscle types. The thermodynamic efficiency of human skeletal muscle was reported as 40% at $20\,{\kern 1pt}^\circ {\text{C}}$ (He et al. 2000). Cardiac muscle from various species exhibited thermodynamic efficiencies of around 20% (Barclay & Loiselle 2020; Barclay et al. 2003). The efficiency of vascular smooth muscle (rabbit rectococcygeus) has been reported as 18% (Walker et al. 1994). Our model predicts a decline in efficiency at lower adverse pressure differences when there is less of a mechanical challenge to overcome. LMCs contain a large number of mitochondria (Ohhashi 1987), so even small losses per mitochondria can sum up to cause losses comparable to cross-bridge cycling. By comparison, metabolic efficiency has been estimated as 80% for cardiac muscle; 4 ×  the cross-bridge cycling efficiency (Barclay & Loiselle 2020; Barclay et al. 2003). Calculating the efficiency of lymphatic pumping is useful for a complete understanding of the regulatory mechanisms, particularly the effects of shear. The two main potential reasons for shear-induced relaxation are that (1) contractions decrease diameter, thereby increasing resistance to flow and (2) energy expenditure of contractions is unnecessary when there is passive flow. Greater understanding of the regulation of lymphatic contractions may yield potential treatments to promote flow in lymphedema. It has been shown experimentally (Gasheva et al. 2006) that shear-reduced tone and stronger phasic contractions result in more energy-efficient pumping.

This was the first study (experimental or computational) to test the effects of varying amplitude and frequency of calcium oscillations in LMCs. Controlling these experimentally would be challenging, so the model presents a more tractable means of exploring the potential effects on pumping. Oscillations cause an increase in flow when strong enough to open the downstream valve, and this effect is stronger as frequency increases. Increased frequency also increases the AP frequency (not modelled here) for more phasic contractions as another means of increasing outflow. Contractile force generation should scale more-or-less linearly with the number of cells contracting in response to calcium oscillations, which is encompassed in the varying of amplitude. Fluctuations in diameter and pressure have been observed experimentally in rat mesenteric lymphatics (Dixon et al. 2006; Dixon ei al. 2005; Gashev et al. 2002; Zhang et al. 2007). It is, however, not confirmed that these fluctuations corresponded to calcium fluctuations and this is still a matter of debate. Experimental observations of the effects of variations in frequency and amplitude of these oscillations would be difficult to obtain in a controlled manner. A commonly theorized cause of these oscillations is spontaneous transient depolarizations (STDs), though this is still the matter of some debate. Inhibitors and activators of STDs used for investigations in smooth muscle (for example inhibitors or blockers of inositol 1,4,5-triphosphate receptors or calcium-activated chloride channels (von der Weid et al. 2008)) could be applied to rat mesenteric lymphatics. However, studying the direct effects on diameter, pressure, and flow fluctuations would be complicated by the lack of action potentials triggered by STDs.

The implementation of this model and demonstration of its function was facilitated by the use of a single lymphangion. It is certainly worthwhile to expand it into series, branched and confluence arrangements of lymphangions, and we have such work underway. Such expansions in scope must be undertaken carefully, given the computational complexity of the multiscale coupling. It is possible that one could simplify the sliding filament model from partial differential equations to ordinary differential equations using the distribution-moment approximation originally developed for striated muscle (Zahalak 1981, 1986) with minimal loss of in vivo fidelity. This approximation assumes a Gaussian displacement-distribution (other distributions could be assumed) of myosin heads to allow for the simplification of the differential equations and causes some additional error in force and velocity approximates but significantly reduces the computational cost. The distribution-moment approximation for the HHM model of smooth muscle has also been developed (Rampadarath 2018). Initial simulations of the distribution-moment model for a single lymphangion have been run but are beyond the scope of this paper.

Calcium regulation by shear stress, diameter, and pressure conditions is not included in our model. LMC calcium regulation reduces contractile frequency at lower pressure differences, causing the LMCs to spend less time in the contracting state, potentially increasing the long-time-averaged efficiency. The increase in diameter that results from fewer contractions plus a notable relaxation in tone reduces resistance to pumping by upstream lymphangions. A future development will be to include calcium regulation with shear stress-, diameter- and voltage-dependent terms to represent calcium fluxes through different channels, following models of various smooth muscles (Bursztyn et al. 2007; Laforêt et al. 2011; Yang et al. 2003). The finding of a range of optimal troponin binding properties should be explored further for potential interventions to increase pumping in lymphedema. While the Hill function for calcium binding to troponin and calmodulin captures the in vivo reality (sigmoidal calcium concentration-isometric force relationship Dougherty et al. (2014)), the assumption of direct proportionality between calcium saturation and transition rates is a potential limitation.

Sliding filament models have inaccuracies in energetics (Bagshaw 1993), particularly during stretching of active muscle (included in our model during relaxation) and shortening at high velocity. The deviation in sliding filament estimates of energy liberation may be due to the tight coupling of ATP hydrolysis and cross-bridge cycling (i.e., one hydrolysis per powerstroke) (Yanagida et al. 1985). It has also been suggested that not every powerstroke hydrolyzes ATP (Yanagida et al. 1985). There have been several models of skeletal muscle which aim to improve energetics estimates by including the weak coupling of ATP hydrolysis and powerstroke cycles. These models included additional binding states (Eisenberg et al. 1980; Piazzesi & Lombardi 1995) or additional rates (two attachment and two detachment) in the two-state model (Barclay 1999). Additional states and pathways were not included in this model because there are no experimental data on lymphatic muscle energetics, so the additional complexity was deemed unnecessary for a first approximation. In future developments of the model, more head states could be included to model weak ATP powerstroke coupling. This will introduce more rate parameters which, due to the lack of experimental data, will be difficult to accurately estimate. Experimental measurements of ATP consumption and muscle heat would be informative.

Cross-bridge overlap dependence is not included in the model, but could be through length-dependent variations in the number of myosin heads that are available for attachment following (Kocková & Cimrman 2009; Zahalak & Motabarzadeh 1997). A first required step would be to distribute the over all overlap dependence of LMCs among the phasic and tonic CEs. Attributing the overlap dependence entirely to the phasic CEs would be justified because of the physiologically low shortening velocity of tonic CEs enforced by the tonic dashpot. Another possible further development would be to model the structural rearrangement of contractile filaments in response to length changes. We could perform something similar to (Brook & Jensen 2014) with an instantaneous change in the number of available heads. Unfortunately, there are no experimental data on which to base such a model or determine its parameters. A further improvement would be to incorporate shifting of the force–length relationship of phasic CEs based on the behavior of the tonic CEs. Imaging capable of distinguishing the phasic and tonic CEs and their overlap would be useful for understanding the interactions of the two contractile types.

In conclusion, we have developed a model of the LMC that provides a first estimate of the energetics and efficiency of LMCs. In development of the model, some structural insights were obtained from the necessity of including a phasic spring and tonic dashpot to obtain physiologic contractions. The model is a flexible basis that could be adapted to study a wide range of aspects of LMC contraction, but a method to perform direct experiments on LMCs would be highly beneficial.

## Supplementary Information

Below is the link to the electronic supplementary material.

Supplementary file1 (DOCX 798 KB)
