# Supplementary material for: A multiscale sliding filament model of lymphatic muscle pumping
Source: Biomech Model Mechanobiol. 2021 Sep 2;20(6):2179–202. doi: 10.1007/s10237-021-01501-0 (PMC8595193; doi:10.1007/s10237-021-01501-0)
Supplement: Supplementary file 1 — Supplementary file1 (DOCX 798 KB) [file 10237_2021_1501_MOESM1_ESM.docx]

# Supplementary material

## Parameter values

Table 5 Lymphangion inputs for comparison with other lymphangion model versions

| **Symbol** | **Description** | **Value [units]** | **Source** |
| --- | --- | --- | --- |
| $p_{e}$ | External pressure | 2 [cmH_2_O] | (Jamalian et al., 2016) |

Table 6 Other parameters

| **Symbol** | **Description** | **Value [units]** | **Source** |
| --- | --- | --- | --- |
| Lymphangion | | |  |
| $\mu$ | Dynamic viscosity of lymph | 0.01 [g/(cm s)] | (Jamalian et al., 2016) |
| $L$ | Lymphangion length | 0.3 [cm] | (Jamalian et al., 2016) |
| $R_{V,min}$ | Open valve resistance | 2.68e6 [g/(cm^4^ s)] | (Jamalian et al., 2017) |
| $R_{V,max}$ | Additional resistance on valve closure | 9e9 [g/(cm^4^ s)] | (Jamalian et al., 2016) |
| $\Delta P_{open}$ | Pressure difference for valve opening | -15 [dyne/cm^2^] | (Jamalian et al., 2016) |
| $\Delta P_{fail}$ | Pressure difference for valve failure | -18032 [dyne/cm^2^] | (Jamalian et al., 2016) |
| $s_{open}$ | Valve opening slope (resistance change for change in pressure difference) | 0.4 [cm^2^/dyne] | (Jamalian et al., 2016) |
| $s_{fail}$ | Valve failure slope (resistance change for change in pressure difference) | 0.049 [cm^2^/dyne] | (Jamalian et al., 2016) |

## Solution flowcharts


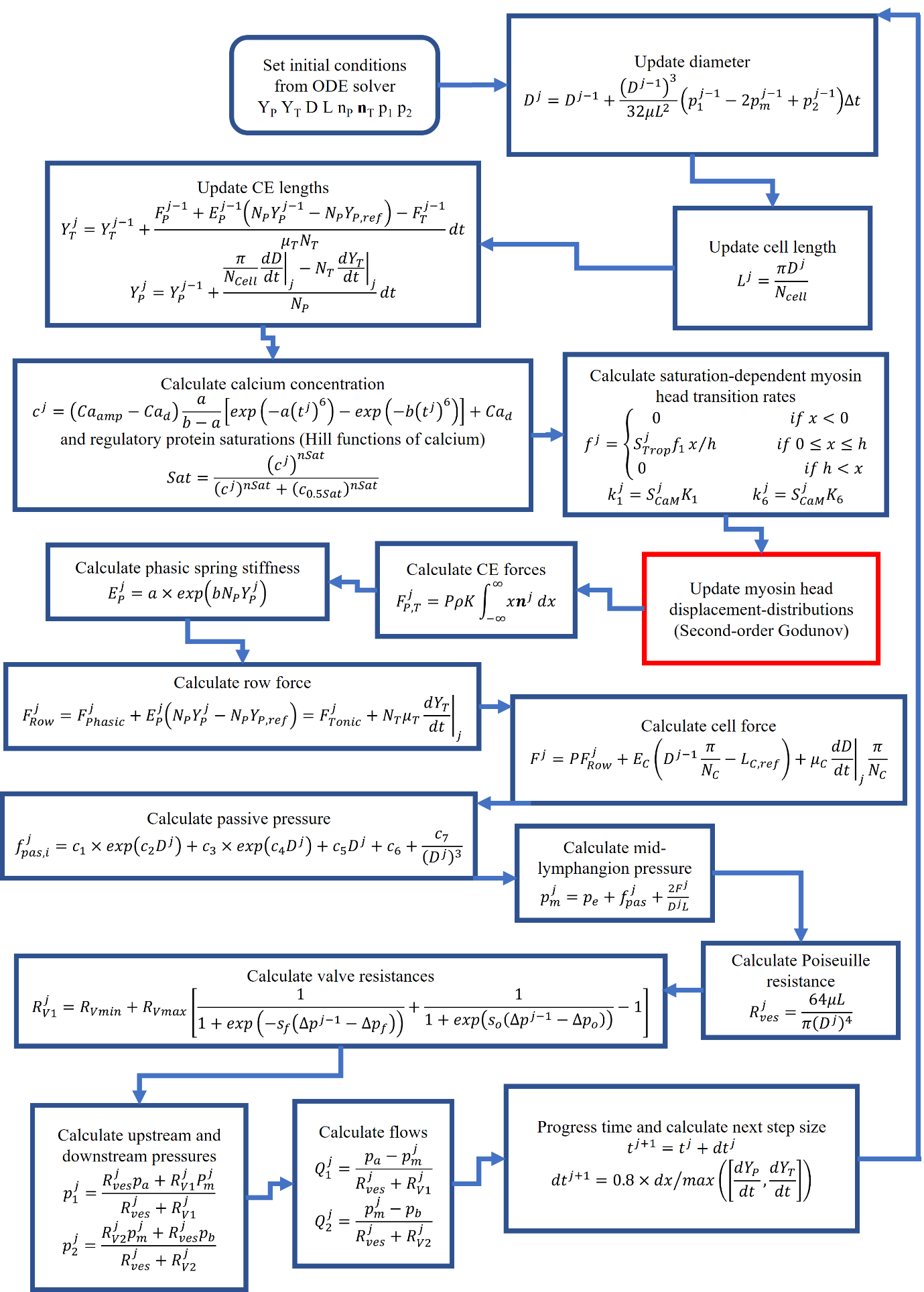


Fig. 12 Flowchart of the solution algorithm for the coupled muscle-lymphangion model. $\boldsymbol{j}$ indexes the time step. The red box indicates use of a Godunov solver (algorithm in Fig. 13)


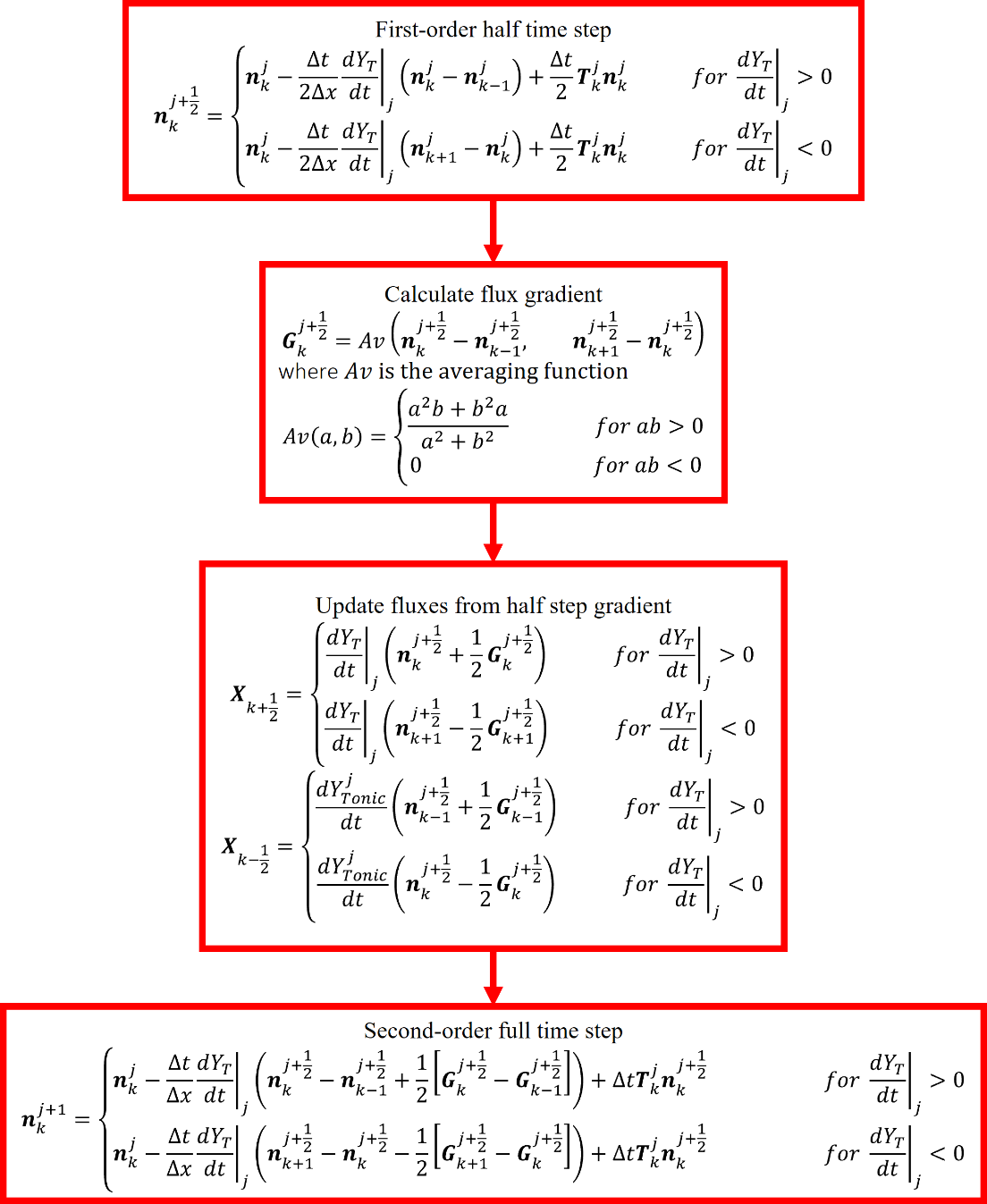


Fig. 13 Solution flowchart for the second-order Godunov solver. $\boldsymbol{k}$ indexes the discretized displacement value

## List of symbols

$a$ Constitutive parameter for strain-stiffening phasic stiffness

$a_{2}$ Constitutive parameter for time-dependent calcium concentration

$AM$ Fraction of tonic heads attached and dephosphorylated

${AM}_{p}$ Fraction of tonic heads attached and phosphorylated

$b$ Constitutive parameter for strain-stiffening phasic stiffness

$b_{2}$ Constitutive parameter for time-dependent calcium concentration

$c$ Intracellular free calcium concentration

$c_{0.5CaM}$ Calcium concentration for calmodulin half saturation

$c_{0.5Trop}$ Calcium concentration for troponin half saturation

${Ca}_{amp}$ Amplitude of action potential calcium increase

${Ca}_{d}$ Diastolic calcium concentration

$cycletime$ Duration of a contractile cycle

$D$ Lymphangion diameter

$D_{0}$ Lymphangion diameter with no intrinsic contractions

$dx$ Size of displacement discretization

$E_{Cell}$ LMC stiffness

$E_{CSloss}$ Energy lost due to cell viscosity

$E_{fluid}$ Useful energy transferred to lymph

$E_{lymph}$ Energy lost due to lymph viscosity

$E_{P}$ Phasic spring stiffness

$E_{TDloss}$ Energy lost due to tonic dashpot viscosity

$EF$ Ejection fraction

${Energy}_{P}$ Energy liberated by detachment of phasic heads

${Energy}_{T,PhosDetach}$ Energy liberated by detachment of phosphorylated tonic heads

${Energy}_{T,UnphosDetach}$ Energy liberated by detachment of unphosphorylated tonic heads

${Energy}_{T,DetachPhos}$ Energy liberated by phosphorylation of detached tonic myosin heads

${Energy}_{T,AttachPhos}$ Energy liberated by the phosphorylation of attached myosin heads

${Energy}_{T}$ Total energy liberated by tonic myosin heads

$F$ Force generated by LMCs

$f$ Phasic attachment rate

$f_{1}$ Maximum phasic attachment rate

$F_{P}$ Phasic CE force

$f_{pas}$ Passive mechanics of lymphangion wall

$F_{Row}$ Force generated by a row of CEs

$F_{T}$ Tonic CE force

$g$ Phasic detachment rate

$g_{1}$ Maximum phasic detachment rate for positive displacements

$g_{2}$ Phasic detachment rate for negative displacements (constant)

$h$ Powerstroke length

$k$ Number of parameters in Latin hypercube sensitivity analysis

$k_{1}$ Phosphorylation rate for detached tonic heads

$k_{2}$ Dephosphorylation rate for detached tonic heads

$k_{3}$ Attachment rate for phosphorylated tonic heads

$k_{4}$ Detachment rate for phosphorylated tonic heads

$k_{5}$ Dephosphorylation rate for attached tonic heads

$k_{6}$ Phosphorylation rate for attached tonic heads

$k_{7}$ Detachment rate for dephosphorylated tonic heads

$K_{3,1}$ Maximum attachment rate for phosphorylated tonic heads

$K_{4,1}$ Detachment rate constant for phosphorylated tonic heads in powerstroke region

$K_{4,2}$ Detachment rate constant for phosphorylated tonic heads at negative displacement

$K_{4,3}$ Additional detachment rate constant for phosphorylated tonic heads at large positive displacement

$K_{7,1}$ Detachment rate constant for dephosphorylated tonic heads in powerstroke region

$K_{7,2}$ Detachment rate constant for dephosphorylated tonic heads with negative displacement

$K_{7,3}$ Additional detachment rate constant for dephosphorylated heads at large positive displacement

$K_{1}$ Rate constant for phosphorylation of detached tonic heads

$K_{2}$ Rate constant for dephosphorylation of detached tonic heads

$K_{5}$ Rate constant for dephosphorylation of attached tonic heads

$K_{6}$ Rate constant for phosphorylation of attached tonic heads

$K_{P}$ Stiffness of phasic heads

$K_{T}$ Stiffness of tonic heads

$L$ Length of a lymphangion

$L_{Cell}$ LMC length

$L_{Cell,ref}$ Reference LMC cell length (zero cell stiffness force)

$M$ Fraction of tonic heads detached and dephosphorylated

$M_{P}$ Fraction of tonic heads detached and phosphorylated

$n$ Number of parameters in LMC model

$N_{Cell}$ Series number of circumferential muscle cells

$n_{mCaM}$ Calmodulin Hill coefficient

$n_{mTrop}$ Troponin Hill coefficient

$N_{P}$ Series number of phasic CEs

$n_{P}$ Fraction of phasic heads attached

$N_{Rows}$ Number of parallel CE rows

$N_{T}$ Number of tonic CEs

${Num}_{P}$ Number of myosin heads in a phasic CE

${Num}_{T}$ Number of myosin heads in a tonic CE

${Osc}_{Amp}$ Amplitude of calcium oscillations

$p_{ext}$ External pressure

$p_{a}$ Inlet pressure boundary condition

$p_{b}$ Outlet pressure boundary condition

$p_{m}$ Mid-lymphangion pressure

$PRCC$ Partial rank correlation coefficient

$Q_{1}$ Lymph flow through first valve

$Q_{2}$ Lymph flow through second valve

$R_{V,max}$ Additional resistance on valve closure

$R_{V,min}$ Open valve resistance

$S$ Outflow sensitivity to parameter values

$S_{CaM}$ Calmodulin saturation

$s_{fail}$ Slope of valve failure (resistance against pressure-difference)

$s_{open}$ Slope of valve opening (resistance against pressure-difference)

$S_{Trop}$ Saturation of troponin

$t$ Time

$t_{Osc}$ Time into current contraction cycle for calcium oscillation onset

$t_{P}$ Time since beginning of current contraction cycle

$u$ Energy liberated by hydrolysis of one ATP molecule

$v_{P}$ Phasic CE shortening velocity

$v_{T}$ Tonic CE shortening velocity

$W$ Work done by LMCs

$x$ Myosin head displacement

$X$ Input values for Latin hypercube sensitivity analysis

$Y$ Model output (average flow) for Latin hypercube sensitivity analysis

$Y_{P}$ Length of a phasic CE

$Y_{P,ref}$ Reference length of phasic CEs (zero phasic spring force)

$Y_{T}$ Length of a tonic CE

$\Delta P_{fail}$ Pressure difference for valve failure

$\Delta P_{open}$ Pressure difference for valve opening

$\mu$ Lymph viscosity

$\mu_{T}$ Tonic dashpot viscosity

$\mu_{Cell}$ LMC viscosity

$\rho$ Linear density of myosin heads

$\omega_{Osc}$ Frequency of calcium oscillations

## Periodicity verification

The simulation for reference conditions was initially run until periodicity conditions were met and was then run for an additional five cycles to check that there was negligible change. The main model outputs of diameter, pressure, and flow did not change (Figure 12 a-d). There is still some variation in tonic CE force and length but they are small (Figure 12 e,f see axes).


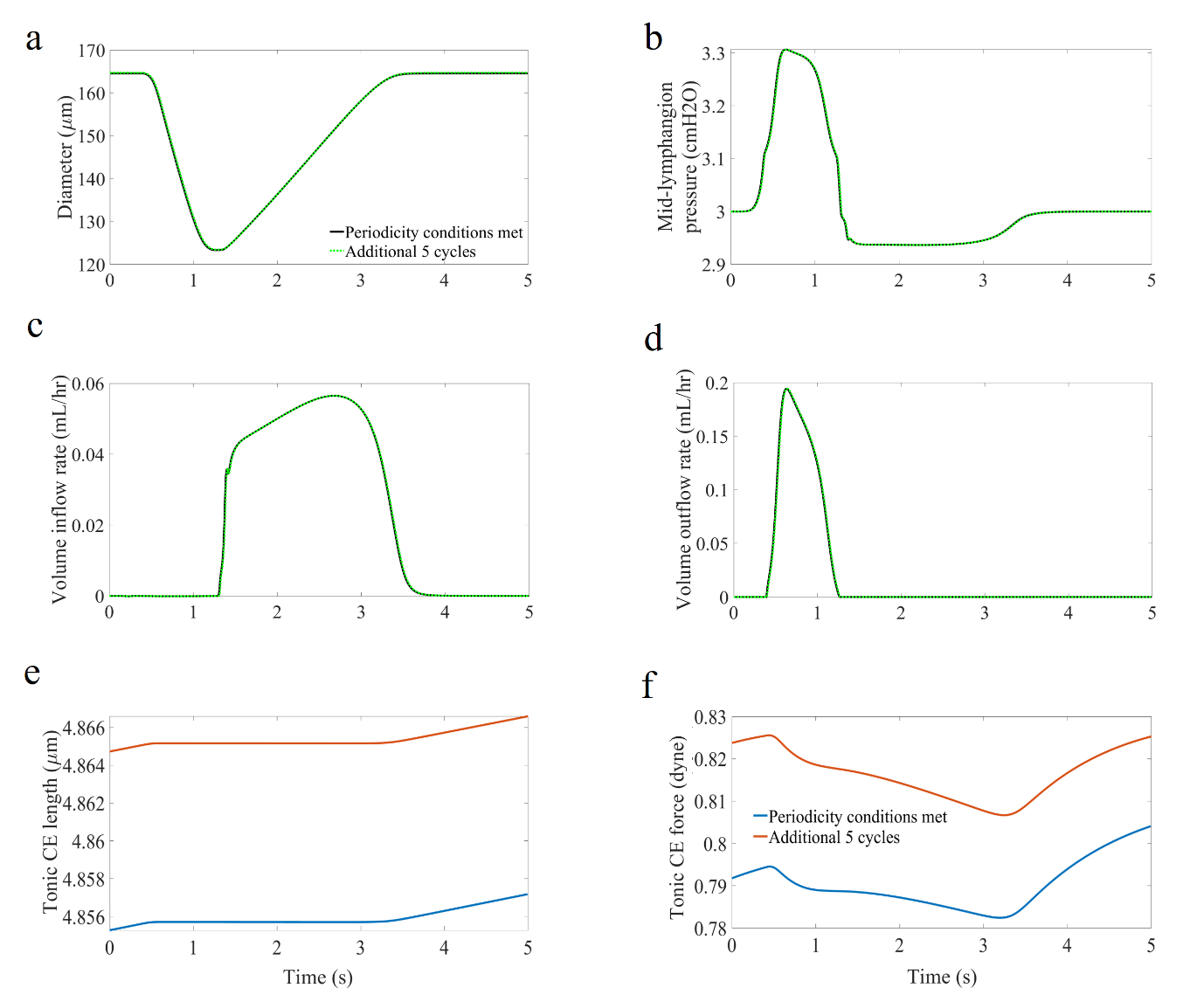


Fig. 14 Panel of plots verifying that the periodicity conditions ensure that the results have reached periodicity. (a) shows that the diameter change is negligible (b) shows that the pressure change is negligible (c) shows that the change in inflow is negligible (d) shows that the outflow rate is negligible (e) shows that there is some variation in tonic CE force and (f) shows that there is some variation in tonic CE length

## Insensitivity to displacement discretisation

Decreasing the displacement discretization from $h/20$ to $h/40$ had a negligible effect on the results under reference conditions. Average flow differed between the two discretisations by only $0.01\%$. The efficiency of muscle was $9.3\%$ and the efficiency of transfer of muscle work to lymph was $30.2\%$ in both cases.


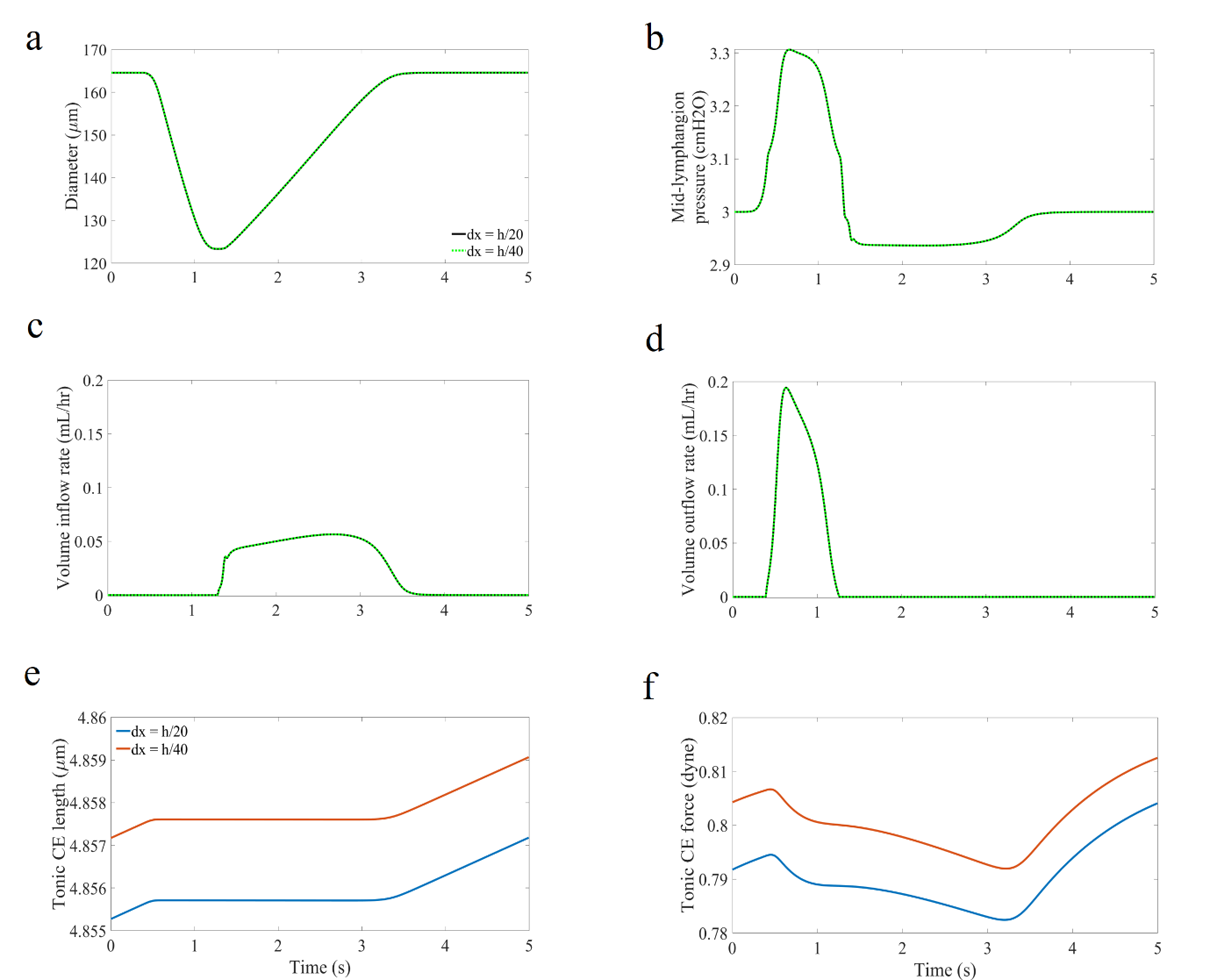


Fig. 15 Panel of plots showing that halving the displacement discretization had a negligible effect on the main output parameters of the model

## One-at-a-time sensitivity analysis results

Table 7 Table of results showing the sensitivity measures obtained for each parameter from the one-at-a-time sensitivity analysis. Rows in bold indicate the variables considered sensitive and included in the Latin hypercube analysis

| **Parameter** | **Input values** | $\boldsymbol{S}$ |
| --- | --- | --- |
| $a_{2} [s^{-6}]$ | 12.085 48.34 120.85 | 0.1909 0.0844 0.0450 |
| $a_{P} [dyne/cm]$ | 5.1282e-24 1.0256e-22 5.1282e-22 | -0.0188 -0.0061 -0.0024 |
| $b_{2} [s^{-6}]$ | 0.2639 1.0556 2.639 | -0.3899 -0.1839 -0.1010 |
| $b_{P} [1/cm]$ | 73.8382 738.3821 | -0.0274 -0.0301 |
| $\boldsymbol{Ca}_{\boldsymbol{amp}}\boldsymbol{[}\mathbf{M}\boldsymbol{]}$ | **1.2e-7 4.8e-7 1.2e-6** | **2.0067 0.5065 0.0003** |
| ${Ca}_{d} [M]$ | 7e-8 2.8e-7 7.0e-7 | 0.6196 -1.0000 -0.2500 |
| $E_{Cell} [dyne/cm]$ | 7.5 150 750 | 0.0594 -0.6729 -0.1032 |
| $f_{1} [1/s]$ | 62 1240 6200 | 0.6050 -0.6009 -0.1061 |
| $K_{3,1} [1/s]$ | 0.088 1.76 8.80 | 1.0e-04 *  0.3037 -0.3800 -0.0467 |
| $g_{1} [1/s]$ | 5 100 500 | 1.0910 -0.0231 -0.0186 |
| $K_{7,1} [1/s]$ | 0.01 0.2 1 | 1.0e-03 *  0.0444 -0.1320 -0.0433 |
| $\boldsymbol{g}_{\boldsymbol{2}} [1/s]$ | **21 420 2100** | **2.4213 0.4602 0.0142** |
| $K_{4,1} [1/s]$ | 0.022 0.44 2.2 | 1.0e-04 *  0.4355 -0.3182 -0.0313 |
| $h [cm]$ | 7.8e-7 3.12e-6 7.8e-6 | 1.0e-05 *  -0.4358 -0.5938 -0.7047 |
| $K_{1} [1/s]$ | 0.035 0.7 3.5 | -0.0134 0.0121 0.0013 |
| $K_{2} [1/s]$ | 0.01 0.2 1 | -0.0134 0.0121 0.0013 |
| $K_{HHM} [dyne/cm]$ | 0.18 3.6 18 | 1.0e-04 *  0.2936 -0.4351 -0.1296 |
| $K_{Hux} [dyne/cm]$ | 0.04 0.8 4 | 0.6402 -0.6034 -0.1064 |
| $m []$ | 3 12 30 | -0.9598 -0.3994 -0.1799 |
| $\mu_{Cell} [dyne s/cm]$ | 5 10 25 | -0.0258 -0.0256 -0.0252 |
| $\mu_{T} [dyne s/cm]$ | 1 20 100 | -0.0122 -0.0008 -0.0002 |
| $\boldsymbol{N}_{\boldsymbol{Cell}}\boldsymbol{[]}$ | **3 5 10** | **3.9802 0.7651 0.6196** |
| $N_{Tonic} []$ | 7 28 50 | 0.0320 0.0151 0.0094 |
| $N_{Phasic} []$ | 7 28 50 | 0.8798 0.6108 0.3853 |
| $\boldsymbol{c}_{\boldsymbol{0.5,CaM}} \mathbf{[M]}$ | **4e-6 20e-6** | **1.0e-04 ***  **0.2782 0.0389** |
| $\boldsymbol{n}_{\boldsymbol{mCaM}}\boldsymbol{[]}$ | **0.5 1 2** | **1.0e-03 ***  **0.7910 0.2688 -0.0802** |
| $N_{Rows} []$ | 1.8e3 3.6e4 1.8e5 | 0.6141 -0.6034 -0.1064 |
| ${Num}_{P} []$ | 450 9000 45000 | 0.6402 -0.6034 -0.1064 |
| ${Num}_{T} []$ | 100 2000 10000 | -0.0220 -0.0071 -0.0026 |
| $\rho[1/cm]$ | 6e4 1.2e6 6e6 | 0.6402 -0.6034 -0.1064 |
